# Chemical and structural characterization of interstrand cross-links formed between abasic sites and adenine residues in duplex DNA

**DOI:** 10.1093/nar/gkv174

**Published:** 2015-03-16

**Authors:** Nathan E. Price, Michael J. Catalano, Shuo Liu, Yinsheng Wang, Kent S. Gates

**Affiliations:** 1 Department of Chemistry, University of Missouri, 125 Chemistry Building, Columbia, MO 65211, USA; 2 Environmental Toxicology Graduate Program, University of California-Riverside, Riverside, CA 92521-0403, USA; 3 Department of Chemistry, University of California-Riverside, Riverside, CA 92521-0403, USA; 4 Department of Biochemistry, University of Missouri, 125 Chemistry Building, Columbia, MO 65211, USA

## Abstract

A new type of interstrand DNA–DNA cross-link between abasic (Ap) sites and 2′-deoxyadenosine (dA) residues was recently reported, but the chemical structure and properties of this lesion were not rigorously established. Here we characterized the nucleoside cross-link remnant released by enzymatic digestion of duplex DNA containing the dA-Ap cross-link. A synthetic standard was prepared for the putative nucleoside cross-link remnant 6 in which the anomeric carbon of the 2-deoxyribose residue was connected to the exocyclic *N*^6^-amino group of dA. Liquid chromatography-tandem mass spectrometry (LC-MS/MS) analysis showed that the synthetic material **6** matched the authentic cross-link remnant released by enzymatic digestion of cross-linked DNA. These findings establish the chemical structure of the dA-Ap cross-link released from duplex DNA and may provide methods for the detection of this lesion in cellular DNA. Both the nucleoside cross-link remnant **6** and the cross-link in duplex DNA were quite stable at pH 7 and 37°C, suggesting that the dA-Ap cross-link could be a persistent lesion with the potential to block the action of various DNA processing enzymes.

## INTRODUCTION

Chemical modification of cellular DNA can have serious biological consequences including cytotoxicity, mutagenesis, carcinogenesis, neurodegeneration and aging (1–8). The chemical structure of a DNA lesion ultimately determines the exact nature of the dysfunction it causes. Thus, careful chemical structure determination is a critical step in the overall characterization of any particular DNA lesion. Rigorous chemical structure determination of DNA adducts typically requires spectroscopic characterization of the lesion (9). This may involve direct characterization of the lesion in, or released from, duplex DNA (10–14). Alternately, a synthetic standard of the putative lesion can be prepared and spectroscopically compared to the authentic lesion released from duplex DNA by enzymatic digestion (9,15–19).

**Scheme 1.**
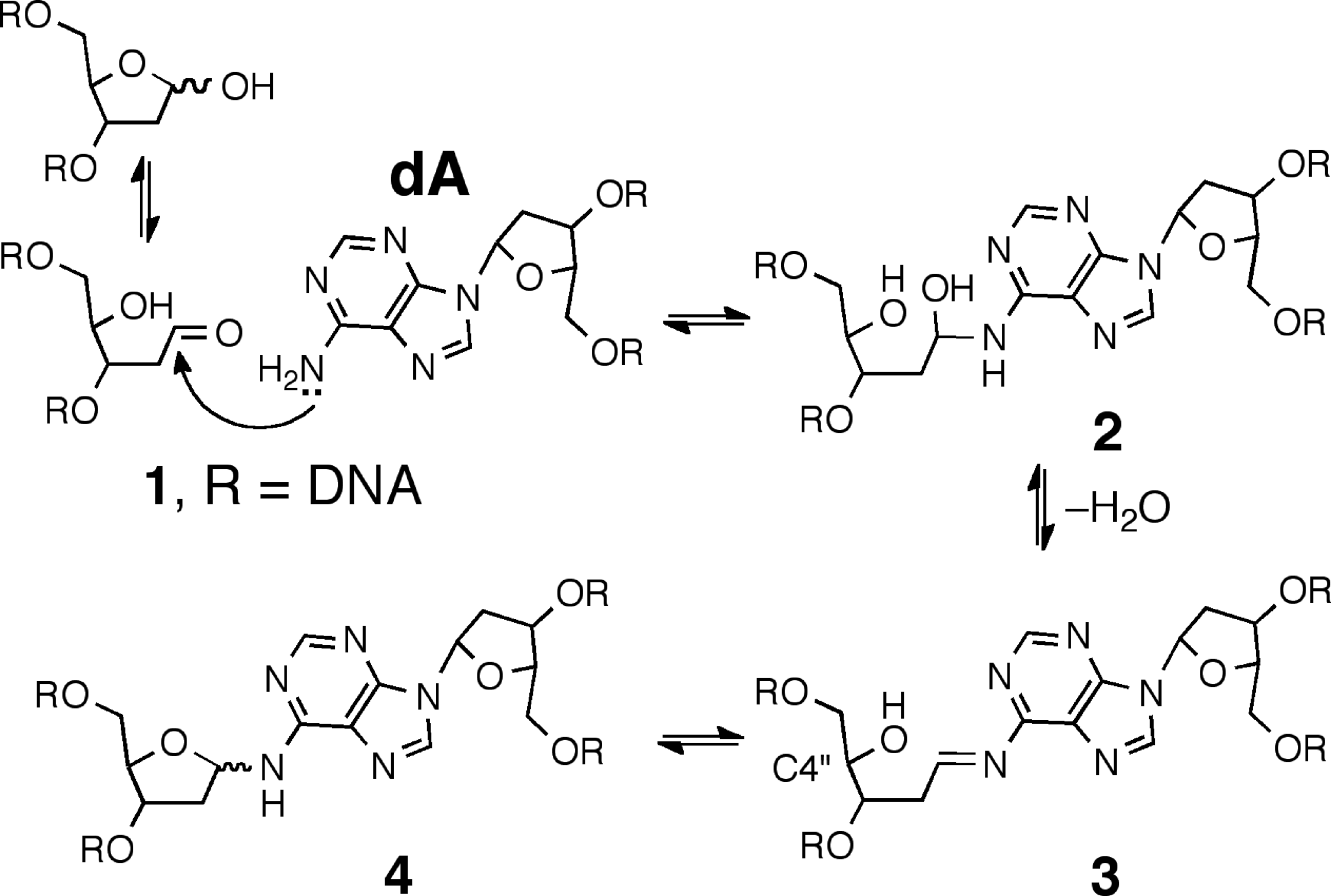
Proposed formation mechanism and structure of the dA-Ap cross-link in duplex DNA.

We recently reported a new type of interstrand DNA–DNA cross-link formed by reaction of a DNA abasic site (Ap site) with a 2′-deoxyadenosine (dA) residue on the opposing strand of the double helix (20). These interstrand cross-links form in high yields (15–70%) at 5′ApT/5′AA sequences in duplex DNA. In our earlier work, we proposed that formation of dA-Ap cross-links involved reaction of the exocyclic *N*^6^-amino group of 2′-deoxyadenosine with the aldehyde residue of the Ap site to generate a cyclic aminoglycoside (**4**, Scheme 1) (21–24). Digestion of double-stranded DNA containing the dA-Ap cross-link with a mixture of nuclease P1, alkaline phosphatase and phosphodiesterases I and II released a nucleoside cross-link ‘remnant’; however, the precise chemical connectivity of the dA-Ap cross-link was not rigorously established.

**Scheme 2.**
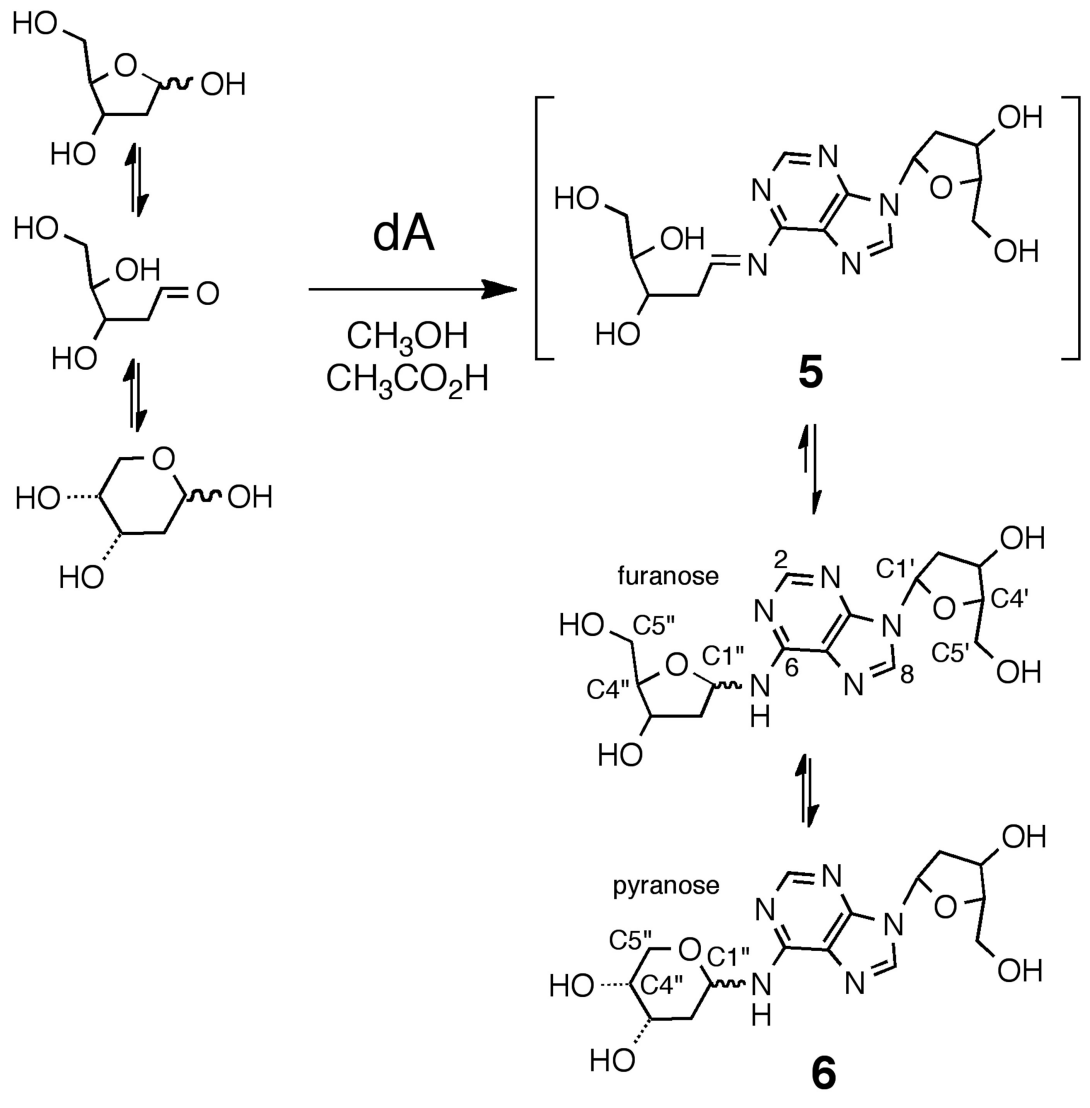
Synthesis of the cross-link remnant **6**.

Here we set out to characterize the chemical structure and properties of the dA-Ap cross-link generated in duplex DNA. Our experimental strategy involved characterization of the cross-link remnant released by enzymatic digestion of DNA duplexes containing the dA-Ap cross-link. We first synthesized an authentic standard of the putative nucleoside cross-link remnant **6** by reaction of dA with 2-deoxyribose (Scheme 2). Complete spectroscopic structure elucidation of this synthetic material rigorously established that the anomeric center of the 2-deoxyribose adduct was connected to the exocyclic *N*^6^-amino group of dA and showed that the compound exists as an equilibrating mixture of the α-pyranose, β-pyranose, α-furanose and β-furanose isomers. Liquid chromatography-tandem mass spectrometry (LC-MS/MS) experiments demonstrated that the properties of the authentic cross-link remnant released by enzymatic digestion of DNA duplexes containing the dA-Ap cross-link matched those of the synthetic material **6**. Overall, these findings established the chemical structure of the dA-Ap cross-link released from duplex DNA and provide a means for the detection of this lesion in cellular DNA. We examined the chemical stability of the cross-link attachment in both the nucleoside remnant (**6**) and in duplex DNA. The cross-link remnant **6** was very stable at neutral pH, decomposing to release unmodified dA with a half-life of 65 days at 37°C. In DNA, the dA-Ap cross-link dissociated with half-lives of 84 and 66 h in duplexes **A** and **B**, respectively. The stability of the dA-Ap cross-link in duplex DNA forecasts the potential for this lesion to block the action of DNA processing enzymes involved in transcription and replication.

## MATERIALS AND METHODS

### Reagents and equipment

Oligonucleotides were purchased from Integrated DNA Technologies (Coralville, IA, USA), uracil DNA glycosylase (UDG, 5000 units/ml) and UDG buffer were purchased from New England Biolabs (Ipswich, MA, USA), 2′-deoxyadenosine (dA) monohydrate was purchased from Tokyo Chemical Industry Co., Ltd. (Tokyo, Japan) and 2-deoxy-d-ribose (99%) was purchased from Sigma-Aldrich (St. Louis, MO, USA). HPLC grade methanol, dichloromethane and H_2_O were purchased from Fisher. All other reagents used were purchased from Acros Organics in reagent grade. Flash column chromatography was performed on silica gel 60 (Sigma-Aldrich). Glass-backed silica gel thin-layer chromatography (TLC) plates impregnated with fluorescent indicator were purchased from Sigma-Aldrich and stored in a desiccator. Compounds were visualized on developed TLC plates by their absorbance using a 254-nm UV lamp or by staining with a solution of 1% KMnO_4_. Nuclear magnetic resonance (NMR) spectra were obtained using a Bruker Avance 800 MHz at 298 K in DMSO-(*d_6_*) or D_2_O (Cambridge Isotope Laboratories, Inc., Cambridge, MA, USA).

### Preparation of cross-linked DNA duplexes

DNA duplexes containing Ap sites at defined locations were prepared using standard procedures (25,26). The 2′-deoxyoligonucleotides (4 nmol) were placed in 150 μl of MOPS buffer (25 mM, pH 7) containing NaCl (100 mM). The duplexes **A** and **B** were annealed by warming the 2′-deoxyuridine-containing oligonucleotide samples to 95°C for 5 min with 3 equivalents of their respective complementary strands, followed by slow-cooling to room temperature overnight in an insulated container. Ap sites were generated by incubation of the DNA duplexes **A** or **B** with UDG (4 μl, 400 units/ml) in a mixture composed of UDG buffer (30 μl) and H_2_O (96 μl) at 37°C for 2 h. Following incubation, UDG was removed by phenol/chloroform extraction. DNA in the aqueous layer was precipitated by mixing with 10% v/v of 3-M aqueous sodium acetate buffer (pH 5.2), followed by 5 vol of absolute ethanol, cooling in dry ice (20 min) and centrifugation for 30 min at 5°C to precipitate the DNA. The pellets were washed twice with 80% ethanol-H_2_O (2 × 180 μl) and resuspended in H_2_O (100 μl). The cross-linking reaction was carried out at 37°C for 120 h in HEPES buffer (50 mM, pH 7.0) containing NaCl (100 mM). After incubation, DNA in the samples was precipitated as described above and loaded onto a 2-mm thick, 20% denaturing polyacrylamide gel and electrophoresed at 200 V for 4 h. The slow-moving, cross-link band was visualized by UV-shadowing and excised from the gel. The gel slice was crushed and the DNA eluted by agitation in 1 ml of aqueous NaCl (200 mM) containing ethylenediaminetetraacetic acid (1 mM, pH 8) for 1 h. The DNA-containing solution was filtered to remove gel fragments, the DNA precipitated, washed and stored at –20°C.

### Enzymatic digestion of cross-linked duplexes

Duplexes **A** and **B** were digested using a 4-enzyme cocktail following the procedures described previously (20,27,28). Briefly, nuclease P1 (5 U), phosphodiesterase 2 (0.01 U), *erythro*-9-(2-hydroxy-3-nonyl)adenine (EHNA, 20 nmol) and a 50-μl solution containing 300-mM sodium acetate (pH 5.6) and 10-mM zinc chloride were added to 300 pmol of duplexes **A** or **B** in a final volume of 500 μl. In this context, EHNA served to inhibit the deamination of 2′-deoxyadenosine (dA) to 2′-deoxyinosine (dI) that can be caused by adenine deaminase impurities in the digestive enzymes (20,27,28). The resulting mixture was incubated at 37°C for 48 h. To the digestion mixture were then added alkaline phosphatase (10 U), phosphodiesterase 1 (0.005 U) and 100 μl of 0.5-M Tris-HCl buffer (pH 8.9) and the digestion continued at 37°C for 2 h. Digestion with nuclease P1 alone was performed according to published procedures (20,27,28), where nuclease P1 (0.2 U) was added to 300 pmol of cross-linked duplex in 500 μl of water. The resulting mixture was incubated at 37°C for 2 h. The enzymatic digestion mixture was extracted with chloroform to remove enzymes, the aqueous layer dried on a Speed-vac, the sample reconstituted in water and subjected to LC-MS/MS analyses (20,27,28).

### *N*^6^-(2-deoxy-d-ribos-1-yl)-2′-deoxyadenosine (**6**)

The compounds 2′-deoxyadenosine monohydrate (154 mg, 0.57 mmol) and 2-deoxy-d-ribose (351 mg, 2.62 mmol) were dissolved in glacial acetic acid (300 μl) and methanol (700 μl). This solvent mixture was used previously for the construction of *N*-aryl aminoglycoside bonds in the synthesis of spicamycin analogs (29). The mixture was incubated at 37°C for 72 h, solvent removed under reduced pressure and the resulting residue subjected to column chromatography on silica gel eluted with a gradient of 5–12% methanol in dichloromethane. The fractions containing product were combined, dried under reduced pressure and evaporated from CDCl_3_ (3 × 2 ml) to yield **6** (115 mg, 54% yield) as a white powder. ^1^H NMR (800 MHz, D_2_O) δ 8.29 (0.63H, s, H8), 8.29 (0.29 H, s, H8), 8.28 (0.08H, s, H8), 8.26 (0.29H, s, H2), 8.25 (0.55H, s, H2), 8.25 (0.08H, s, H2), 8.24 (0.08H, s, H2), 6.43 (1H, t, *J* = 6.9 Hz, dR1), 6.28 (0.08H, br s, H1″), 6.21 (0.08H, br s, H1″), 5.81 (0.29H, brs, H1″), 5.48 (0.55H, brs, H1″), 4.64 (1H, m, H3′), 4.47 (0.08H, m, H3″), 4.44 (0.08H, m, H3″), 4.29 (0.29H, m, H3″), 4.17 (1H, m, H4′), 4.10 (0.08H, m, H4″), 4.07 (0.55H, m, H3″), 4.02 (0.08H, m, H4″), 3.94 (0.55H, m, H5″), 3.90 (0.84H, m, H4″), 3.84 (0.29H, m, H5″), 3.83 (1H, m, H5′), 3.79 (0.55H, m, H5″), 3.77 (1H, m, H5′), 3.76 (0.29H, m, H5″), 3.70 (0.08H, m, H5″), 3.69 (0.08H, m, H5″), 3.65 (0.08H, m, H5″), 3.64 (0.08H, m, H5″), 2.80 (1H, m, H2′), 2.66 (0.08H, m, H2″), 2.55 (1H, m, H2′), 2.39 (0.08H, m, H2″), 2.33 (0.08H, m, H2″), 2.24 (0.29H, m, H2″), 2.16 (0.08H, m, H2″), 2.13 (0.55H, m, H2″), 2.08 (0.29H, m, H2″), 2.07 (0.55H, m, H2″); ^13^C NMR (200 MHz, D_2_O) δ 156.1 (C6), 156.0 (C6), 155.9 (C6), 154.9 (C2), 154.8 (C2), 154.8 (C2), 154.7 (C2), 151.3 (C4), 143.5 (C8), 143.3 (C8), 122.2 (C5), 122.1 (C5), 122.0 (C5), 90.1 (C4′), 90.1 (C4′), 88.6 (C4″), 87.9 (C4″), 87.4 (C1′), 87.3 (C1′), 88.6 (C1″), 80.3 (C1″), 77.9 (C1″), 74.3 (C3″), 73.9 (C3′), 73.8 (C3″), 70.3 (C3″), 69.6 (C5″), 69.3 (C4″), 69.2 (C4″), 68.5 (C3″), 66.1 (C5″), 64.7 (C5″), 64.4 (C5′), 63.9 (C5″), 41.7 (C2″), 41.7 (C2′), 41.6 (C2″), 37.1 (C2″), 35.6 (C2″).

### *N*^6^-[3,5-bis-*O*-methyl-2-deoxy-d-ribofuranos-1-yl]-2′-deoxyadenosine (**7**)

A mixture of 3,5-bis-*O*-methyl-2-deoxy-d-ribofuranose (30,31) (263 mg, 1.62 mmol) and 2′-deoxyadenosine (100 mg, 0.40 mmol) was dissolved in 2 ml of a 1:1 (v/v) mixture of ammonium phosphate buffer (25 mM, pH 7) and DMSO, stirred at 37°C for 9 days and the mixture evaporated to yield a yellow oil. Column chromatography on silica gel eluted with a gradient of 0–5% methanol in CH_2_Cl_2_ gave **7** as a colorless oil (22 mg, 14%): ^1^H NMR (500 MHz, DMSO-*d*_6_) δ 8.45–8.39 (1H, m, H8), 8.35–8.00 (1.5H, m, H2, *N*^2^-H), 7.55 (0.5H, brs, *N*^2^-H), 6.40–6.33 (1H, m, H1′), 6.24 (1H, brs, H1″), 5.35 (1H, brs, 3′-OH), 5.23–5.08 (1H, m, 5′-OH), 4.41 (1H, brs, H3′), 4.07 (0.5H, q, *J* = 4.3, H4″), 3.94–3.89 (0.5H, m, H4″), 3.89–3.85 (1H, m, H4′), 3.85–3.81 (1H, m, H3″), 3.64–3.58 (1H, m, H5a′), 3.55–3.48 (1H, m, H5b′), 3.36–3.30 (3.5H, m, H5″, *O*-CH_3_), 3.26 (1.5H, s, *O*-CH_3_), 3.25 (1.5H, s, *O*-CH_3_), 3.24 (1.5H, s, *O*-CH_3_), 2.71 (1H, ddd, *J* = 13.4, 6.6, 6.6, H2a′), 2.43–2.33 (0.5H, m, H2a″), 2.33–2.22 (1.5H, m, H2b′, H2a″), 2.20–2.08 (1H, m, H2b″); ^13^C NMR (126 MHz, DMSO-*d*_6_) δ 153.5 (C6), 152.1 (C2), 149.1 (C4), 140.3, 140.1 (C8), 119.6 (C5), 88.0 (C4′), 83.8 (C1′), 81.7 (C3″), 81.5 (C3″), 80.9 (C4″), 80.6 (C4″, C1″), 73.3, 72.8 (C5″), 70.8 (C3′), 61.8, 61.7 (C5′), 58.5, 56.8, 56.0 (*O*-CH_3_), 39.4, (C2′), 35.8 (C2″). TOF MS(ES+): 396.1881.

### *N*^6^-[1-deoxo-2-deoxy-3,5-bis-*O*-methyl-d-ribofuranos-1-yl]-2′-deoxyadenosine (**8**)

Compound **7** (18 mg, 0.05 mmol), NaBH_3_CN (100 mg, 1.6 mmol) and acetic acid (25 μl, 0.4 mmol) were dissolved in dry methanol (5 ml). The mixture was warmed to 40°C and stirred for 48 h, at which time additional portions of NaBH_3_CN and acetic acid were added (200 mg, 3.2 mmol and 50 μl, 0.8 mmol, respectively). After 68 h, the reaction was mixed with saturated aqueous Na_2_CO_3_ (5 ml) and the resulting suspension lyophilized. The resulting residue was brought back up in methanol, filtered to remove solids and the filtrate evaporated to give a colorless oil. Column chromatography on silica gel eluted first with ethyl acetate and then with CH_2_Cl_2_-methanol (9:1) gave **8** as a colorless oil (8 mg, 44%, *R*_f_ = 0.21 in 9:1 CH_2_Cl_2_-methanol): ^1^H NMR (500 MHz, DMSO-*d*_6_) δ 8.32 (1H, s, H8), 8.20 (1H, s, H2), 7.80 (1H, brs, *N*-H), 6.34 (1H, dd, *J* = 7.5, 6.5, H1′), 5.31 (1H, d, *J* = 3, 3′-OH), 5.25 (1H, t, *J* = 5.25, 5′-OH), 4.81 (1H, brs, 4″-OH), 4.43–4.38 (1H, m, H3′), 3.88 (1H, q, *J* = 3.3, H4′), 3.67–3.59 (2H, m, H4″, H5a′), 3.59–3.48 (3H, m H1″, H5b′), 3.37–3.31 (1H, m, H5a″), 3.29 (3H, s, *O*-CH_3_), 3.28–3.25 (1H, m, H5b″), 3.23 (3H, s, *O*-CH_3_) 3.22–3.18 (1H, m, H3″), 2.72 (1H, ddd, *J* = 13.1, 6.6, 6.6, H2a′), 2.25 (1H, ddd, *J* = 13.1, 6.1, 2.6, H2b′), 1.90–1.81 (1H, m, H2a″), 1.77–1.66 (1H, m, H2b″); ^13^C NMR (126 MHz, DMSO-*d*_6_) δ 154.6 (C6), 152.4 (C2), 148.0 (C4), 139.3 (C8), 119.7 (C5), 88.0 (C4′), 84.0 (C1′), 79.8 (C3″), 74.0 (C5″), 71.0 (C3′), 70.2 (C4″), 61.9 (C5′), 58.3, 57.3 (*O*-CH_3_), 39.4 (C2′), 36.8 (C1″), 29.6 (C2″). TOF MS(ES+): 398.2034.

### Analysis of the stability of **6**

Compound **6** was dissolved in sodium phosphate buffer (100 mM, pH 7.0) to give a final concentration of 5 mM and a final volume of 8 ml. The solution was incubated at 37°C and aliquots (300 μl) were removed at specific time points and analyzed by HPLC employing a Varian Microsorb-MV C18 (250 × 4.6 mm) column eluted with water (solvent A) and acetonitrile (solvent B) in the following gradient: 0–5 min 0% B, 5–50 min 0–18% B, 50–55 min 18–0% B and 55–60 min 0% B. Products were detected by their absorbance at 250 nm.

## RESULTS AND DISCUSSION

### Synthesis and spectroscopic characterizations of the cross-link remnant **6**

The putative cross-link remnant **6** was prepared by reaction of 2′-deoxyadenosine (0.6 M) with 2-deoxy-d-ribose (2.6 M) in a solvent mixture-composed methanol-acetic acid (2:1 v/v; Scheme 2). Thin layer chromatographic analysis of the reaction mixture on silica gel revealed formation of a single new polar spot. Column chromatography gave the product in 54% yield.

**Figure 1.**
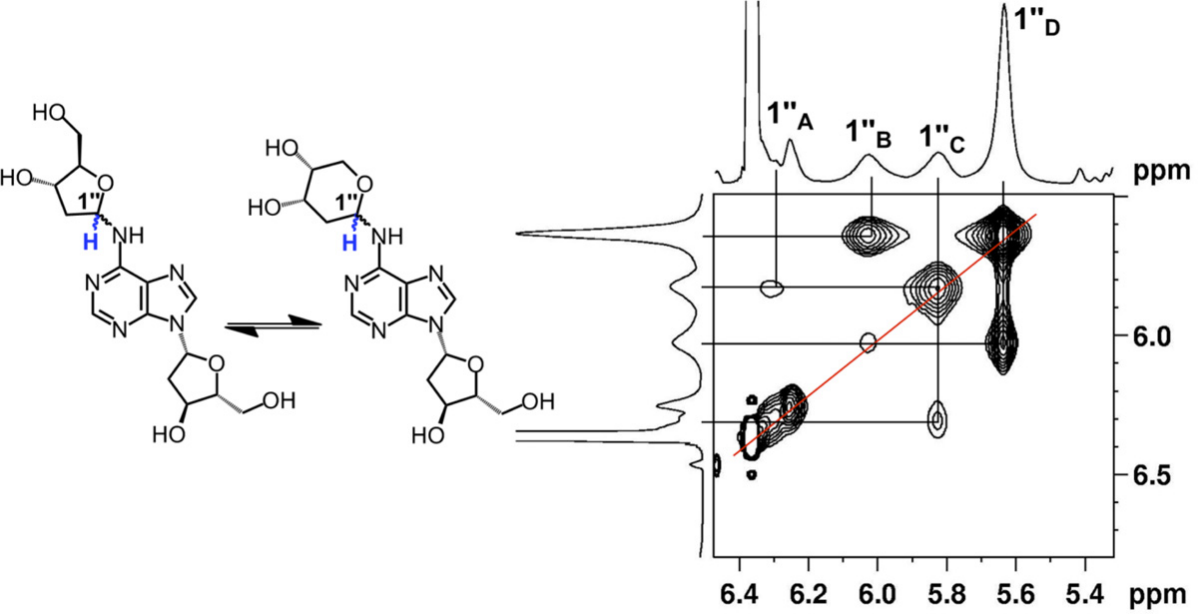
EXSY-2D-NMR of the H1″ spectral region for compound **6**. Correlations between the isomers of **6** appear as contours at the intersecting lines between signals for the H1″ protons, indicating a dynamic equilibrium between the α-pyranose, β-pyranose, α-furanose and β-furanose isomers of the 2-deoxyribose adduct located at the exocyclic *N*^6^-amino group of the adenine residue.

Proton NMR analysis revealed resonances for both dA and an additional 2-deoxyribose unit consistent with the anticipated structure **6** (Tables S1 and S2). The HMQC spectra allowed assignment of carbon-to-hydrogen connectivity. Multiple resonances were observed for the 2-deoxyribose adduct in the product. This was not unexpected, as 2-deoxyribose itself exists as an equilibrium mixture of the α-pyranose, β-pyranose, α-furanose and β-furanose isomers (Scheme 2) (32). Similarly, *N*-aryl-2-deoxyaminoriboside analogs exist as a mixture of cyclic forms, in which the pyranose isomers typically dominate (21,33–36). Thus, the observation of multiple resonances for the carbons and hydrogens of the 2-deoxyribose unit in the product suggested that the adduct was present as an equilibrium mixture of four possible isomers (α-pyranose, β-pyranose, α-furanose and β-furanose). A 2D-EXSY-NMR experiment that gives cross-peaks between sites that are in slow chemical exchange confirmed that the multiple resonances reflected an equilibrium mixture of 2-deoxyribose isomers in the product. For example, the H1″ signals from 5.6–6.3 ppm are a dynamic isomeric mixture of four separate signals (Figure 1).

**Figure 2.**
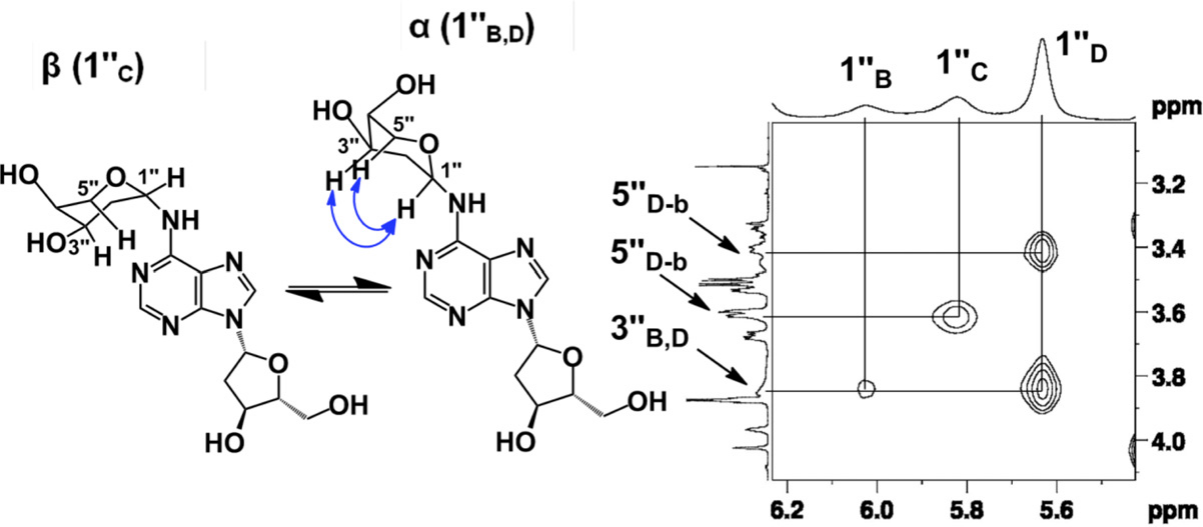
NOESY spectrum of the H1″ resonances of compound **6**. The correlations between the 3″ and 1″ protons suggest that resonances B and D are α-anomers. The absence of this correlation suggests that resonance C is from the β-anomer.

The 2-deoxyribose adduct in **6** has the potential to exist as a ring-opened imine, carbinolamine or a cyclic hydroxyalkylhemiaminal. The observed chemical shifts of the of 75–82 ppm for C1″ are consistent with the cyclic structures **6** (35). The NMR data provided no evidence for imine, carbinolamine or enamine functional groups in the product. The NMR results further suggest that the α-pyranose isomer predominates in solution. This is supported by observation that C4″ in the major isomer displays a chemical shift of 69 ppm (35) and the presence in this isomer of correlations between the C5″ hydrogens and the C1″ carbon in the HMBC experiment. The major isomer showed a strong NOE cross-peak between the H1″ and H3″, consistent with the α-isomer (Figure 2). The ratio of isomers in water was ∼7:4:1:1 (Supplementary Figure S10) which is similar to the ratio of isomers reported for unsubstituted 2-deoxy sugars (37).

**Figure 3.**
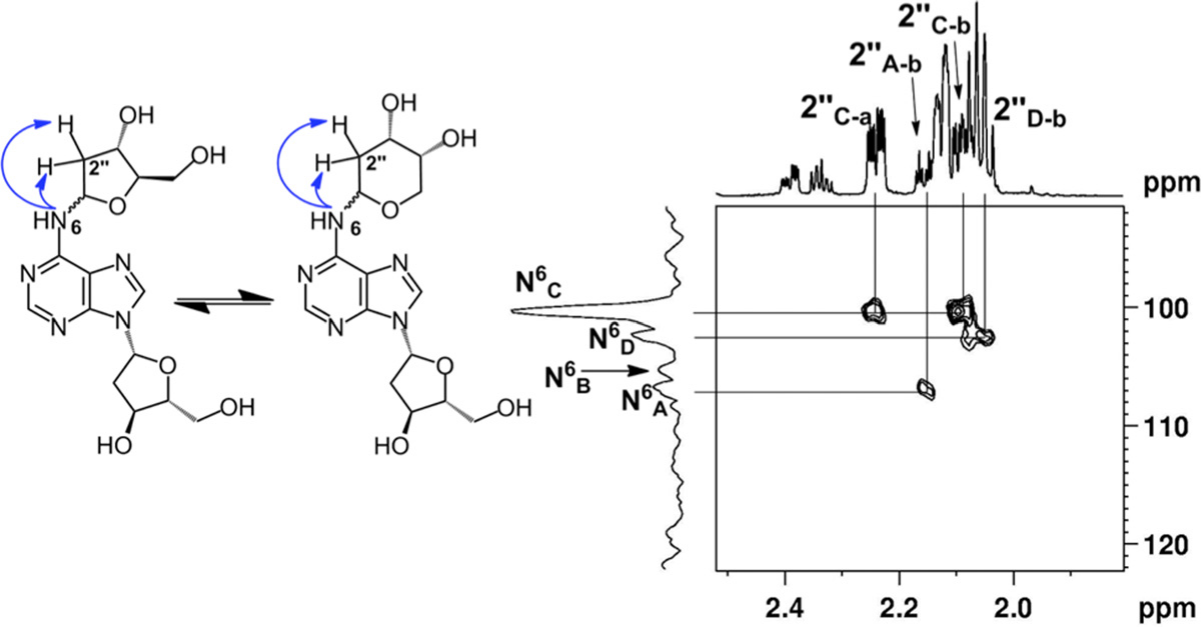
^15^*N*-HMBC data showing the H2″ spectral region of compound **6**. The correlations between *N*^6^ of dA and H2″ of dR can be seen by contours shown at the intersecting lines between signals for the *N*^6^ and H2″ protons, indicating a two to three-bond coupling between these atoms.

**Figure 4.**
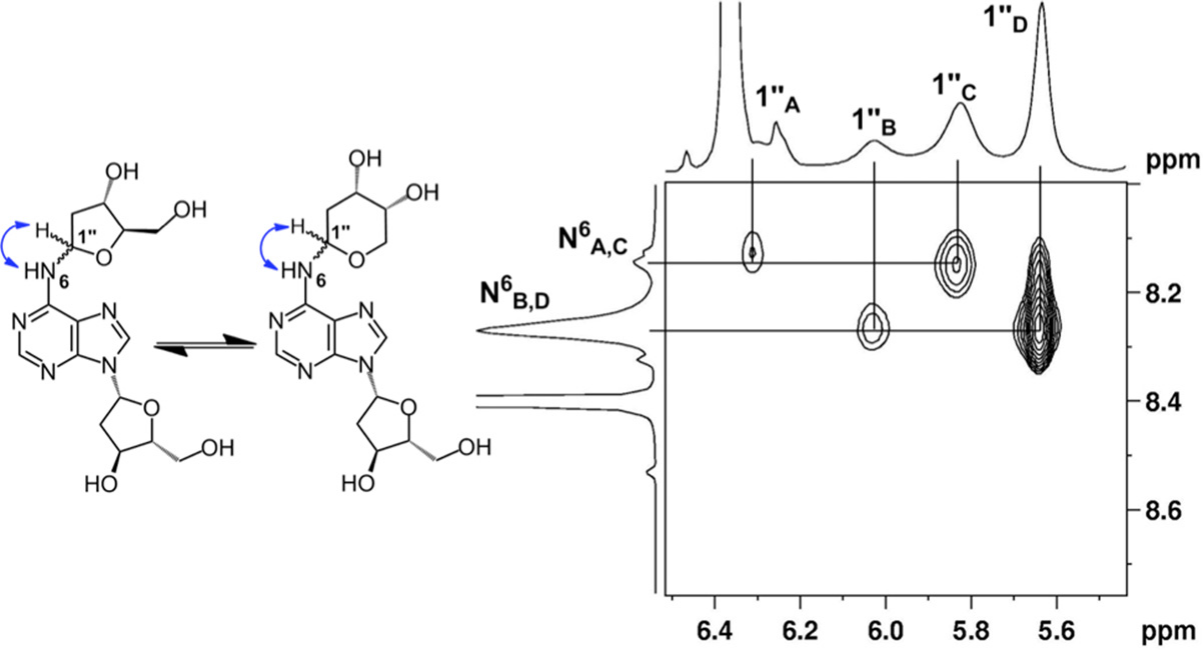
TOCSY data showing the *N*^6^-H correlations with H1″ protons of compound **6**. The correlations between the *N*^6^-H of dA and H1″ of dR can be seen by contours shown at the intersecting lines between signals for the *N*^6^-H and H1″ protons, indicating these protons are in the same spin system.

The NMR data provided evidence that the anomeric carbon of the 2-deoxyribose adduct was attached at the exocyclic *N*^6^-amino group of the 2′-deoxyadenosine unit, as shown in structure **6**. For example, an ^15^N-HMBC experiment revealed three-bond coupling between the *N*^6^-nitrogen and the H2″ protons of the 2-deoxyribose adduct (Figure 3). Furthermore, the TOCSY spectra showed that the *N*^6^-H, the H1″ and H2″ protons reside in the same spin system (Figure 4). Overall, the NMR analysis established that the reaction of dA with 2-deoxyribose provided the desired cross-link remnant **6**.

### Stability of the dA-dR cross-link remnant 6 and the dA-Ap cross-link in duplex DNA

**Figure 5.**
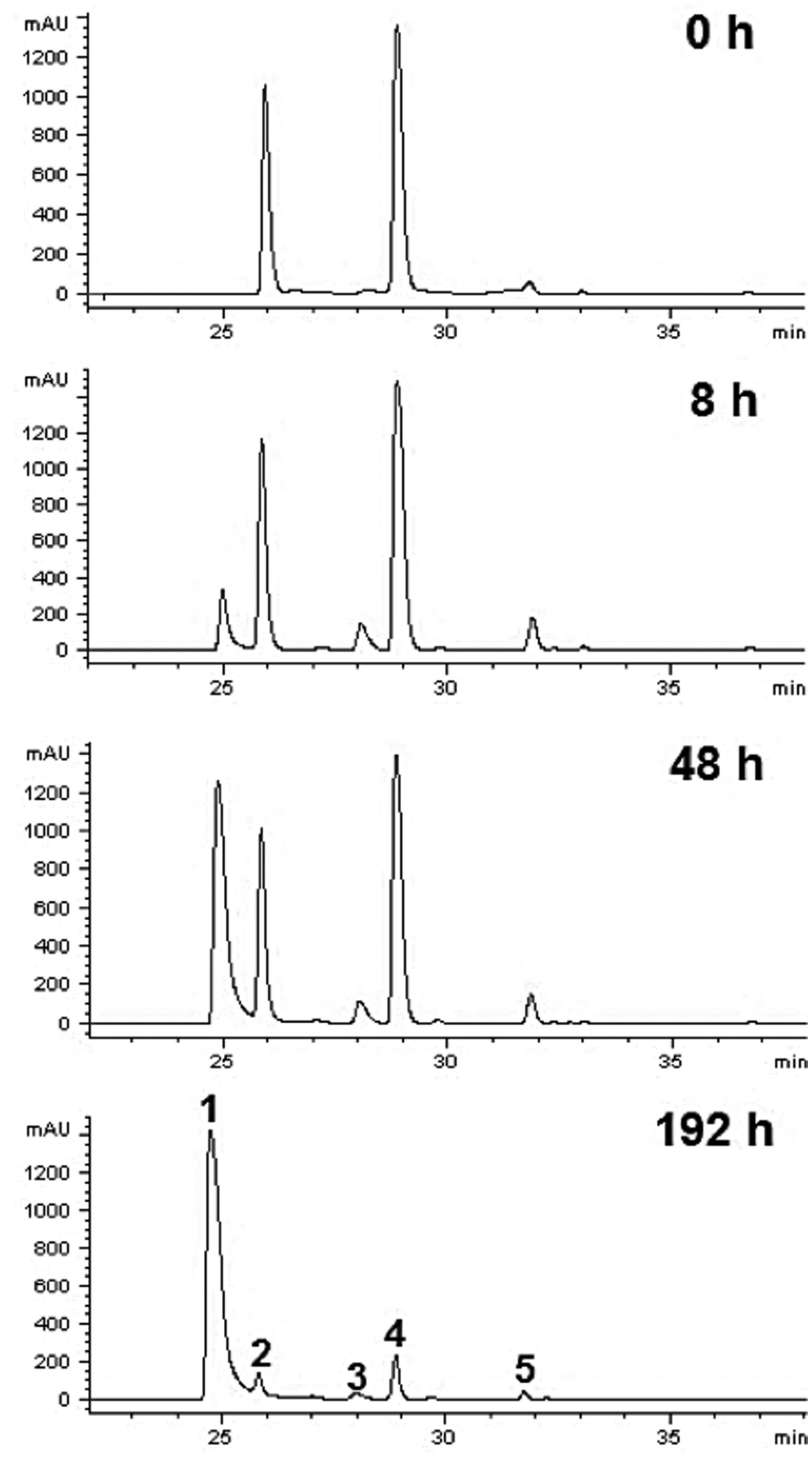
HPLC chromatogram panels for the dissociation of compound **6** at 75°C in 20 mM sodium phosphate (pH 7.0). Peak 1 is 2′-deoxyadenosine, peaks 2 and 4 are major isomers of compound **6** while peaks 3 and 5 are minor isomers of compound **6**. k_37°C_ = 1.05 ± 0.02 × 10^–2^ d^-1^, k_75°C_ = 2.41 ± 0.01 × 10^–1^ d^-1^ (estimated E_a_ = 74 kJ/mol or 18 kcal/mol).

**Figure 6.**
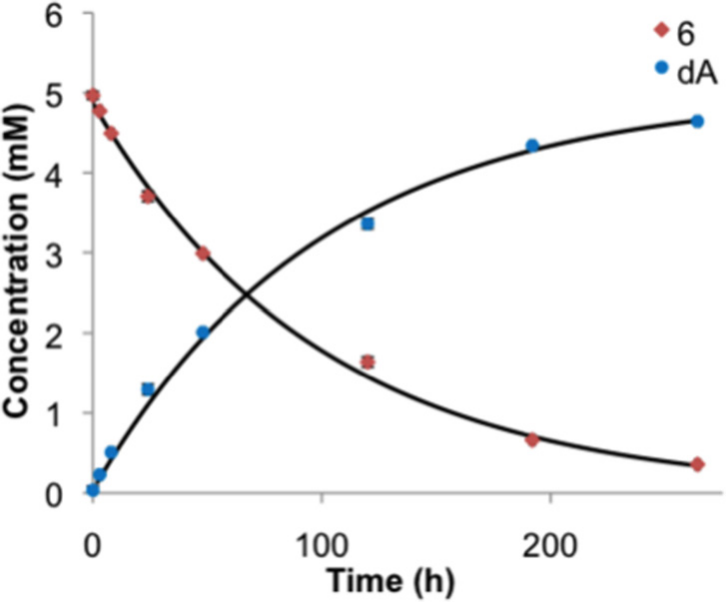
Decomposition of compound **6** (falling curve) at 75°C into 2-deoxyribose and dA (rising curve). HPLC analysis of the products (UV 250 nm) was used to observe the disappearance of **6** and the appearance of dA. The solid lines represent a nonlinear regression analysis fit to a first-order growth or decay process with k_75°C_ = 6.5 × 10^–3^ h^-1^.

Before endeavoring to use the synthetic cross-link remnant **6** as an analytical standard, we examined the chemical stability of this material. Compound **6** can be classified broadly as an *N*-aryl glycosylamine. Compounds of this type have the potential to undergo hydrolytic decomposition to release the free sugar and the unmodified arylamine (38–40). We used HPLC to examine the stability of **6** in sodium phosphate buffer (50 mM, pH 7) at both 37 and 75°C. First, we noted that all four isomers of **6** described above in the context of the NMR experiments could be resolved by reversed-phase HPLC (Figure 5). Second, we found that the synthetic cross-link remnant **6** was remarkably stable, decomposing to release unmodified dA with a half-life of 65 days at 37°C and 3 days at 75°C (Figure 6).

**Figure 7.**
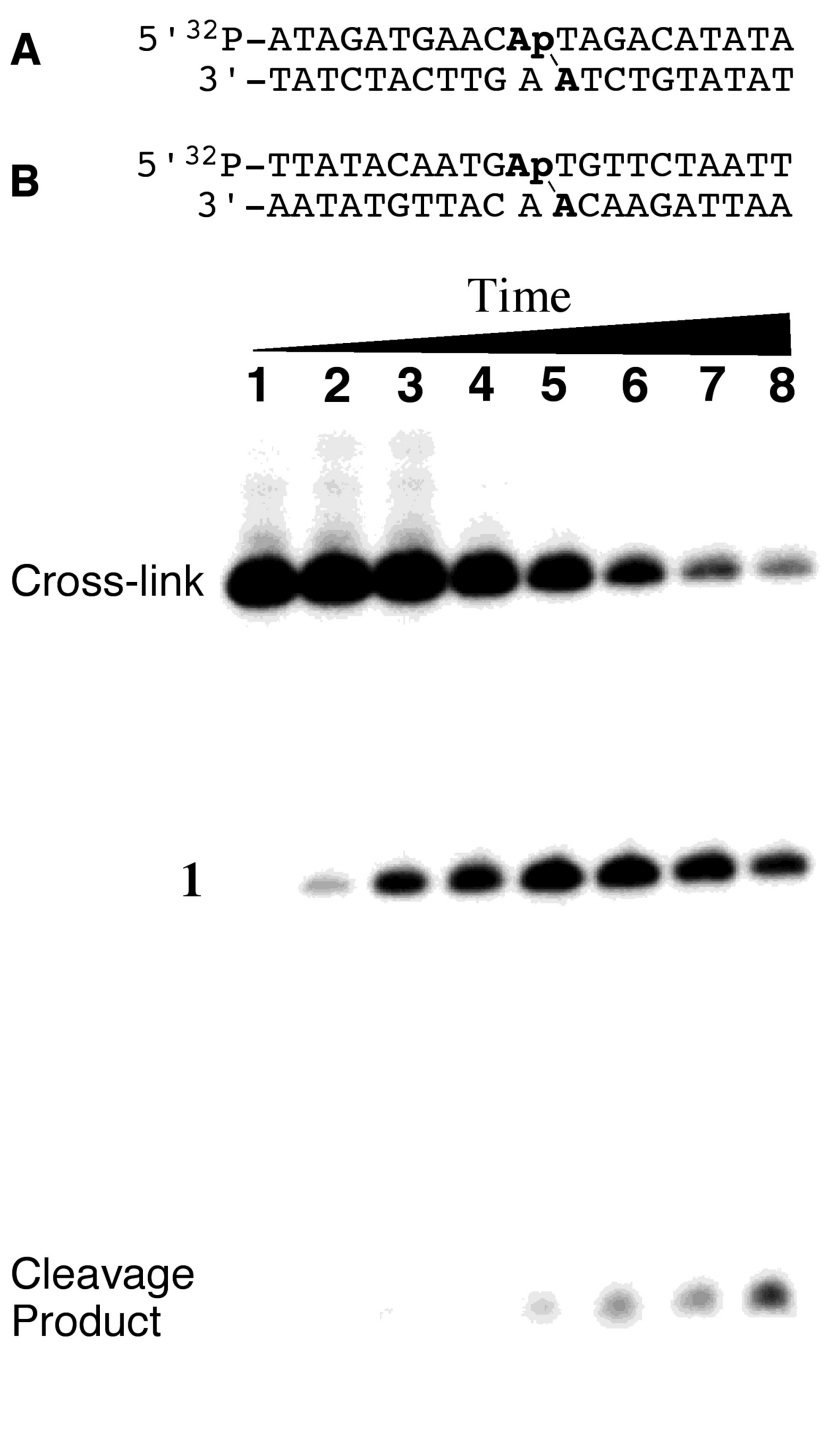
Above: cross-linked oligonucleotide duplexes used for enzymatic digestion and LC-MS/MS analysis and stability studies (duplexes used in mass spectrometric studies did not contain the ^32^P label). Location of the dA-Ap cross-links is indicated with the line (\). Below: dissociation of the purified cross-link in duplex A incubated in 50 mM HEPES (pH 7.0) and 100 mM NaCl at 37°C for 0, 0.25, 1, 2, 5, 10, 15 and 21 days (lanes 1–8). Following incubation for the specified time, DNA in the samples was ethanol precipitated, washed and stored at –20°C until analysis by 20% denaturing gel electrophoresis. The amount of remaining cross-link at each time point was quantitatively measured by phosphorimager analysis.

We also monitored the stability of the dA-Ap cross-link embedded within two different DNA duplexes (Figure 7). The 5′-^32^P-radiolabeled, cross-linked duplexes **A** and **B** were prepared as described previously (20), gel purified and then redissolved in HEPES buffer (50 mM, pH 7) containing NaCl (100 mM) and incubated at 37°C. At various times, aliquots were removed, frozen and later subjected to analysis by denaturing polyacrylamide gel electrophoresis (Figure 7 and Supplementary Figure S20). The dA-Ap cross-links within duplexes **A** and **B** were quite stable, dissociating with half-lives of 66 and 85 h, respectively (Supplementary Figures S20 and S21), to give the Ap-containing oligonucleotide **1** (Scheme 1) and small amounts of the 3′-4-hydroxy-2-pentenal-5-phosphate cleavage product resulting from β-elimination at the Ap site (1,41,42). In a separate experiment, the amount of remaining cross-linked duplexes **A** and **B** was monitored after incubation at 22°C over the course of 96 h, in three different buffers with pH values of 5, 7, or 9. At pH 5, 25 and 40% dissociation of cross-linked duplexes **A** and **B**, respectively, into the component single strands was observed. No dissociation of the cross-linked duplexes **A** and **B** was observed over the course of 96 h at 22°C at pH 7 or 9 (Supplementary Figure S22).

### Evidence that the synthetic nucleoside cross-link remnant **6** matches the authentic remnant released by enzymatic digestion of DNA containing the dA-Ap cross-link

**Figure 8.**
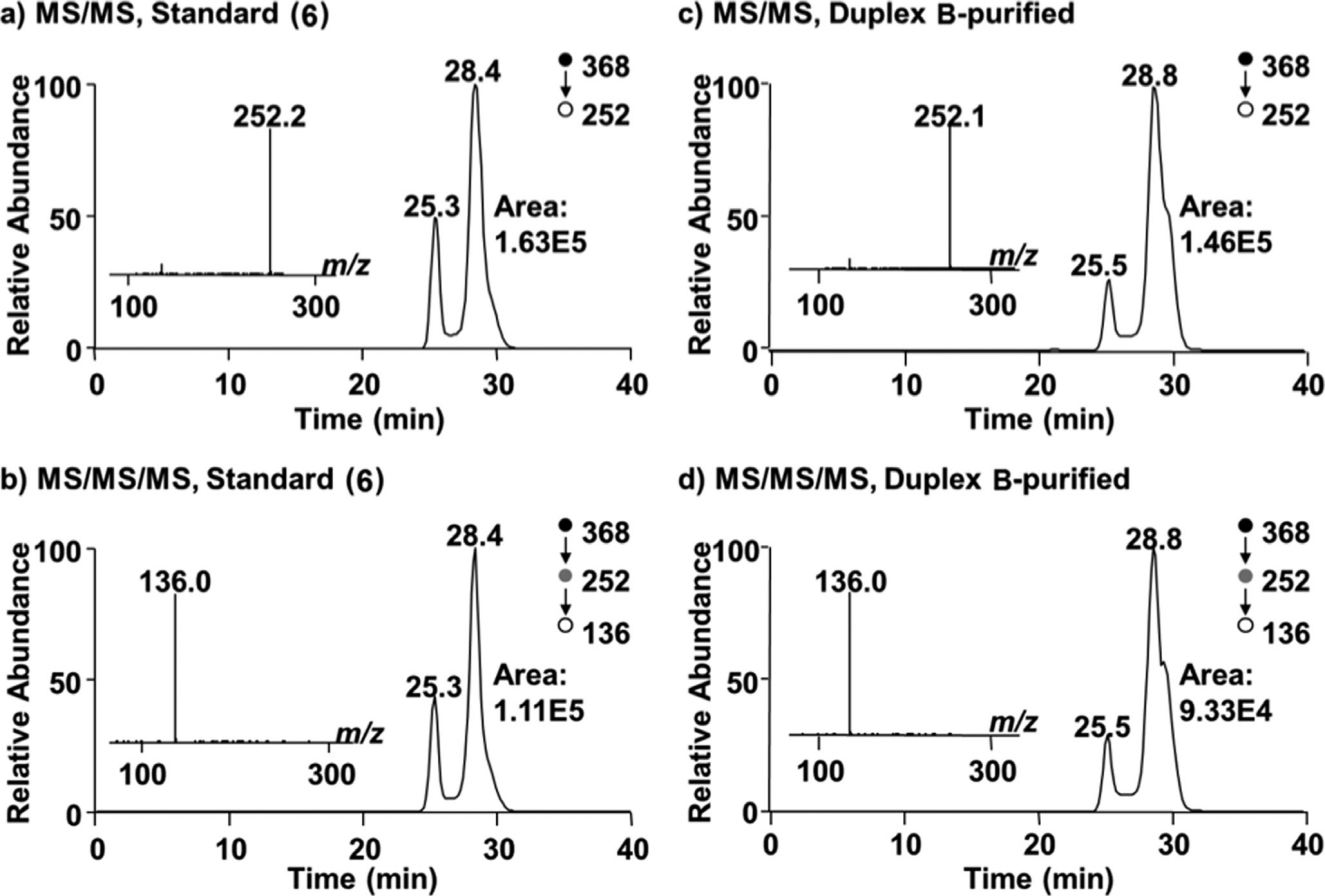
LC-MS/MS and -MS/MS/MS of the synthetic standard **6** and the enzymatically digested cross-linked duplex B. Panels (**a**) and (**b**) are for the synthetic nucleoside **6**, while panels (**c**) and (**d**) show the cross-link remnant obtained from digestion of duplex B. Panels (a) and (c) are the selected-ion chromatograms for the *m*/*z* 368→252 transition. Panels (b) and (d) are the selected-ion chromatograms for the *m*/*z* 368→252→136 transition. Shown in the insets are the MS/MS ((a) and (c)) and MS/MS/MS ((b) and (d)) averaged from the 28.4-min fraction in (a) and (b), and 28.8-min fraction in (c) and (d).

With a synthetic standard of the putative dA-Ap cross-link remnant **6** in hand, we used LC-MS/MS analysis to determine whether this synthetic material matched the actual remnant released from cross-linked DNA by enzymatic digestion. We have previously characterized formation of the dA-Ap cross-link in two different DNA sequence contexts, **A** and **B** (Figure 7) (20). These cross-linked duplexes (lacking the 5′-^32^P-label) were prepared, gel purified and treated with a four enzyme cocktail consisting of nuclease P1, alkaline phosphatase and phosphodiesterases I and II to release the cross-link remnant as previously described (20). A separate control experiment showed that the synthetic standard **6** was stable under the enzymatic digestion conditions (Supplementary Figure S18). The digested oligonucleotide mixtures and the synthetic material **6** were analyzed by LC-MS/MS and -MS/MS/MS, where we monitored the fragmentation of the [M+H]^+^ ion (*m/z* 368) of the cross-link remnant and the further fragmentation of the ion of *m/z* 252 observed in MS/MS, respectively. We found multiple peaks in the selected-ion chromatograms for monitoring the *m/z* 368→252 transition in MS/MS (for the loss of a 2-deoxyribose), and the *m/z* 368→252→136 transition (for the loss of another 2-deoxyribose) in MS/MS/MS. These results are consistent with aforementioned observations that the cross-link remnant exists as an equilibrium mixture of the α-pyranose, β-pyranose, α-furanose and β-furanose isomers that are separable by HPLC. Importantly, the retention times and fragmentation pattern of the synthetic material **6** matched that of the remnant released by enzymatic digestion of the cross-linked duplexes **A** and **B** (Figure 8 and Supplementary Figure S19). These results provide evidence that the chemical connectivity of the dA-Ap cross-link is that shown in structure **6** (Scheme 2).

### Chemical reduction of the cross-link remnant **6**

**Scheme 3.**
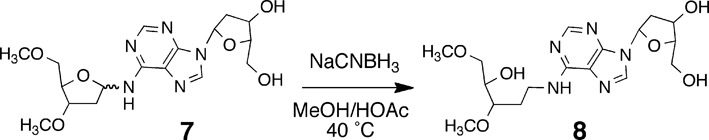
Reduction of the 5′/3′-protected cross-link remnant **7**.

As shown in Schemes 1 and 2, the dA-Ap cross-link is an imine-derived lesion. While our NMR analysis provided evidence that the synthetic cross-link remnant **6** exists predominantly in the ring-closed form, equilibration of the isomers presumably proceeds via small amounts of the imine **5**. Similarly, the hydrolytic decomposition of **6** described above likely proceeds via attack of water on the presumptive imine intermediate **5**. Imine-derived adducts can be stabilized by reduction using reagents such as sodium cyanoborohydride (43–47). Accordingly, we examined whether the cross-link remnant **6** was a substrate for hydride reduction. Interestingly, we were not able to execute reduction of **6** using common hydride donors including sodium borohydride, sodium cyanoborohydride and sodium triacetoxyborohydride. Various solvent mixtures (methanol, water and acetic acid), solution pH values (3–7) and amounts of hydride donor (10–1000 equiv) were examined, but no new product could be detected by TLC, NMR or HPLC analysis. Greenberg *et al*. similarly found that a cross-link derived from the reaction of dA with 5′-(2-phosphoryl-1,4-dioxobutane) in duplex DNA was refractory to reduction by NaCNBH_3_ (48). We suspected that the equilibrium amounts of the reactive imine **5** might be too small to allow a reasonable rate of reduction. By way of analogy, there are examples in which the equilibrium amount of free, ring-opened aldehyde dictates the reaction rates of aldoses (49). To test this idea we synthesized an analog of the dA-dR cross-link remnant that cannot access the favored pyranose isomer due to protection of the 5′- and 3′-hydroxyl groups in the 2-deoxyribose adduct (**7**; Scheme 3 and Supplementary Figures S12–S14). We anticipated that this analog might present a larger fraction of the reactive imine **5**. Indeed, we found that compound **7** can be converted, albeit rather slowly, to the reduced form **8** in 44% yield by reaction with NaCNBH_3_ (100 equiv) and acetic acid (∼ 2% v/v) at 40°C in methanol for 68 h (Scheme 3 and Supplementary Figures S15–S17). Slow reduction of other aldehyde-derived imine adducts on DNA, including a formaldehyde adduct at *N*^6^-dA and FaPy-dG lesions, has been observed by others (35,50,51).

## CONCLUSIONS

This work was designed to shed light on the structure and properties of the dA-Ap cross-link formed in duplex DNA. We carried out a chemical synthesis and structure determination of the putative nucleoside cross-link remnant **6**. LC-MS analysis showed that the properties of the synthetic material matched that of the authentic remnant released from cross-linked DNA by enzymatic digestion. The results provided evidence that the dA-Ap cross-link involves attachment of the exocyclic *N*^6^-amino group of dA to the anomeric carbon of the Ap residue. In addition, our work provides methodology that can be applied to the detection of this cross-link lesion in cellular DNA.

Although the dA-Ap cross-link is an imine-derived lesion (Scheme 1), our spectroscopic evidence indicated that the 2-deoxyribose adduct exists as an equilibrium mixture of the α-pyranose, β-pyranose, α-furanose and β-furanose isomers. No evidence for the ring-opened imine or carbinolamine structures was observed. Cyclization of the sugar hydroxyl residue(s) may serve to mask the hydrolytically labile imine intermediate, thus stabilizing the dA-Ap cross-link. Both the nucleoside cross-link remnant and the cross-link in duplex DNA are stable for days at neutral pH. The stability of the dA-Ap cross-link suggests that this may be a persistent lesion with the potential to block the action of various DNA-binding and DNA-processing enzymes.

## SUPPLEMENTARY DATA

Supplementary Data are available at NAR Online.

SUPPLEMENTARY DATA
