# Supplementary material for: Chemical and structural characterization of interstrand cross-links formed between abasic sites and adenine residues in duplex DNA
Source: Nucleic Acids Res. 2015 Mar 16;43(7):3434–41. doi: 10.1093/nar/gkv174 (PMC4402519; doi:10.1093/nar/gkv174)
Supplement: SUPPLEMENTARY DATA [file supp_43_7_3434__index.html]

Chemical and structural characterization of interstrand cross-links formed between abasic sites and adenine residues in duplex DNA — Chemical and structural characterization of interstrand cross-links formed between abasic sites and adenine residues in duplex DNA — SUPPLEMENTARY DATA 

# Chemical and structural characterization of interstrand cross-links formed between abasic sites and adenine residues in duplex DNA

## SUPPLEMENTARY DATA

**Files in this Data Supplement:**

- SUPPLEMENTARY DATA
