# Supplementary material for: Chemical and structural characterization of interstrand cross-links formed between abasic sites and adenine residues in duplex DNA
Source: Nucleic Acids Res. 2015 Mar 16;43(7):3434–41. doi: 10.1093/nar/gkv174 (PMC4402519; doi:10.1093/nar/gkv174)
Supplement: SUPPLEMENTARY DATA [file supp_gkv174_NP_dA-dR_SI_3_7_15.pdf]

Supplementary Data for:

Chemical and structural characterization of interstrand cross-links formed between abasic sites and adenine residues in duplex DNA

Nathan E. Price[‡], Michael Catalano[‡], Shuo Liu[⊥], Yinsheng Wang[†⊥], and Kent S. Gates[‡,§,*]

[‡]Department of Chemistry, University of Missouri, Columbia, MO 65211
[§]Department of Biochemistry, University of Missouri, Columbia, MO 65211
[†]Department of Chemistry, University of California-Riverside, CA 92521-0403
[⊥]Environmental Toxicology Program, University of California-Riverside, CA 92521-0403

*E-mail: gatesk@missouri.edu, Ph: (573) 882-6763

Table of Contents

Table S1. NMR chemical shifts and correlations for compound **6** in D_2O……………………..…..3

Table S2. NMR shifts and correlations for compound **6** in DMSO-d_6………………………...4

Figure S1. UV absorbance spectra of 2'-deoxyadenosine and **5** ……………………..……... 5

Figure S2. ^{1}H-NMR in deuterium oxide of **5** ..……………………….......................... 6

Figure S3. ^{13}C-NMR in deuterium oxide of **5** ..……………………..…........................ 7

Figure S4. ^{13}C DEPT-NMR of **5** in D_2O...……………………..……………….............. 8

Figure S5. ^{1}H-NMR of **5** in DMSO-(d_6) …………...……………….……........................ 9

Figure S6. ^{13}C-NMR of **5** in DMSO-(d_6) …………...………………..……........................10

Figure S7. ^{15}N-HMBC of **5** in DMSO-(d_6) …………...……………..……........................11

Figure S8. D_2O exchange of N^6-proton in **5** …………...…………..……….................... 12

Figure S9. HMQC of the deoxyribose H2' and H2" spectral region of **5** …………...…......... 13

Figure S10. HMQC of the H1" region of **5** …………...…………………….......................... 14

Figure S11. TOCSY of the N^6-H correlations with H2" of **5** ……………......................... 15

Figure S12. ^{1}H-NMR of **6** in DMSO-(d_6)...……………………….................................... 16

Figure S13. ^{13}C-NMR of **6** in DMSO-(d_6).. 15

Figure S14. COSY showing correlation of N^6-proton with H1” of **6** in DMSO-(d_6)................... 16

Figure S15. ^{1}H-NMR of **7** in DMSO-(d_6).. 17

Figure S16. ^{13}C-NMR of **7** in DMSO-(d_6).. 18

Figure S17. COSY showing correlation of N^6-proton with H1” of **7** in DMSO-(d_6)................... 19

Figure S18. Stability of **5** under conditions of enzymatic digest... 20

Figure S19. LC-MS/MS of compound **5** released from duplex **A** 21

Figure S20. Electrophoretic analysis of stability of cross-link in duplexes **A** and **B** 22

Figure S21. Plots of electrophoretic stability data for cross-link in duplexes **A** and **B** 23

Figure S22. Stability of cross-link in duplexes **A** and **B** at pH 5.2, 7, and 9.2............................ 24

Table 1

Position	Isomer	δ_c		δ_H (J in Hz)	HMBC	TOCSY	^{15}N HMBC
6	A	156.1					
	B	156					
	C	155.9					
2	A	154.9		8.26 0.29H, s	4, 6		
	B	154.8		8.25 0.55H, s	4, 6		
	C	154.8		8.25 0.08H, s	4, 6		
	D	154.7		8.24 0.08H, s	4, 6		
4		151.3					
8	A	143.5		8.29 0.63H, s	4		
	B	143.3		8.29 0.29H, s	4		
	C			8.29*			
	D			8.28 0.08H, s			
5	A	122.2					
	B	122.1					
	C	122.0					
4'		90.1		4.17 1H, m	1'	1', 3', $5'_{a,b}$, $2'_{a,b}$	
4''	A	88.6		4.10 0.08H, m		$5''_{A\text{-}a,b}$	
	B	87.9		4.02 0.08H, m	$5''_{B}$	$5''_{B\text{-}a,b}$	
	C	69.2		3.90 0.90H, m	$2''_{D}$	$2''_{D\text{-}a,b}$	
	D	69.3		3.90 *			
1'		87.4		6.43 1H, t(6.9)	4, 8, 4'	3', 4', $2'_{a,b}$	
1''	A	84.5		6.28 0.08, brs		$3''_{A}$, $2''_{A\text{-}a,b}$	
	B	ND		6.21 0.08H, brs		$3''_{B}$, $2''_{B\text{-}a,b}$	
	C	80.3		5.48 0.55H, brs		$2''_{C\text{-}a,b}$	
	D	77.9		5.81 0.29H, brs		$3''_{D}$, $2''_{D\text{-}a,b}$	
3''	A	74.3		4.47 0.08H, m		$1''_{A}$, $2''_{A\text{-}a,b}$	
	B	73.8		4.44 0.08H, m		$1''_{B}$, $2''_{B\text{-}a,b}$	
	C	70.3		4.07 0.55, m		$1''_{C}$, $4''_{C}$/, $2''_{C\text{-}a,b}$	
	D	68.49		4.29 0.29H, m		$1''_{D}$, $5''_{D\text{-}a,b}$, $2''_{D\text{-}a,b}$	
3'		73.9		4.64 1H, m	1', 5'	4', $5'_{a,b}$	
5''	C	69.6	a	3.94 0.55H, m	$1''_{C}$, $3''_{C}$		
			b	3.79 0.55H, m	$1''_{C}$		
	D	66.1	a	3.84 0.29H, m	$1''_{D}$, $4''_{D}$		
			b	3.76 0.29H, m	$1''_{D}$		
	A	64.7	a	3.70 0.08H, m		$4''_{A}$	
			b	3.65 0.08H, m		$4''_{A}$	
	B	63.9	a	3.69 0.08H, m		$4''_{B}$	
			b	3.64 0.08H, m		$4''_{B}$	
5'		64.4	a	3.83 1H, m	4', 3'	4', 3'	
			b	3.77 1H, m	4'	4'	
2'		41.7	a	2.80 1H, m	4', 1', 3'	1', 4'	
			b	2.55 1H, m	4', 3'	1', 4'	
2''	B	41.6	a	2.66 0.08H, m	$4''_{B}$, $3''_{B}$	$1''_{B}$, $3''_{B}$	
			b	2.16 0.08H, m	$4''_{B}$, $3''_{B}$	$1''_{B}$, $3''_{B}$	
	A	41.6	a	2.39 0.08H, m	$3''_{A}$	$1''_{A}$, $3''_{A}$	
			b	2.33 0.08H, m	$1''_{A}$	$1''_{A}$, $3''_{A}$	
	D	37.1	a	2.24 0.29H, m	$3''_{D}$	$1''_{D}$, $3''_{D}$	
			b	2.08 0.29H, m	$1''_{D}$	$1''_{D}$, $3''_{D}$	
	C	35.6	a	2.13 0.55H, m	$4''_{C}$, $3''_{C}$	$1''_{C}$, $3''_{C}$, $4''_{C}$	
			b	2.07 0.55H, m	$1''_{C}$, $4''_{C}$, $3''_{C}$	$1''_{C}$, $3''_{C}$, $4''_{C}$	
N6	A	106.5					$2''_{A\text{-}b}$
	B	105					
	D	102					$2''_{D\text{-}b}$
	C	100.5					$2''_{C\text{-}a,b}$

* Overlapped by a larger peak

Table 1. NMR chemical shifts and correlations for compound **6** in D_2O.

Table 2

Position	Isomer	δ_c		δ_H (J in Hz)	HMBC	TOCSY	EXSY	NOESY
6		153.8						
2		152.6		8.27 1H, brs	6			
4		149.4						
8		140.6		8.40 1H, s	4, 5, 1'			
5		120.2						
N6	A,C			8.15 0.46H, brs		$2''_{a,b}$, $1''_{C}$		
	B,D			*8.27 0.39H, brs		$2''_{a,b}$, $1''_{B,D}$		
4'		88.4		3.88 1H, m	1', 3', $2'_{a}$	1', 3'-OH, 5'-OH, 3', $5'_{a,b}$		
3''	A,C	86.9		3.68 0.4H, m	$1''_{C}$	$1''_{C}$, $2''_{a,b}$		
	B,D	67.9		3.85 0.5H, m				$1''_{B,D}$
			OH	5.09 1H		$2''_{a,b}$, $3''_{D}$		
1'		84.4		6.36 1H, t (6.9)	8, 4, 4', 3'	3', $2'_{a,b}$		
1''	A	84.2		6.26 0.1H, brs		$N6_{A}$, $2''_{a,b}$	$1''_{C}$	
	B	80.6		6.05 0.2, brs		$N6_{B}$, $3''_{B}$, $2''_{a,b}$	$1''_{D}$	
	C	79.4		5.83 0.3H, brs		$N6_{A}$, $4''_{C}$, $2''_{a,b}$	$1''_{A,D}$	
	D	75.5		5.63 0.4H, brs		$N6_{B}$, $3''_{D}$, $2''_{a,b}$	$1''_{B,C}$	
4''	D	71.7		4.16 0.4H, m		$2''_{a,b}$		
	A,B	67.3		4.09 0.3H, brs				
	C	66.9		3.97 0.3H, m	$3''_{C}$, $5''_{A\text{-}b}$, $2''_{a}$	$1''_{C}$		
			OH	4.62 1H	$4''_{B}$, $2''_{a}$	$4''_{C}$		
3'		71.3		4.40 1H, m	1', 4', $5'_{a}$	1', 3'-OH, 4', $5'_{a,b}$, $2'_{a,b}$		
			OH	5.30 1H	4', 3', $2'_{a}$	1', 3', 4', $2'_{a,b}$		
5''	D,B	65.7	a	3.48 0.6H, m	$4''_{D}$			
			b	3.41 0.6H, m	$1''_{D}$			$1''_{B,D}$
	C,A	64.9	a	**3.61 0.4H, m	$4''_{A}$, $5''_{A\text{-}b}$, $3''_{A}$			$1''_{C}$
			b	**3.51 0.4H, m	$4''_{A}$			
			OH	5.01 1H	$4''_{A}$			
5'		62.2	a	3.61 1H, m	4', 3'	5'-OH, 4'		
			b	3.52 1H, m	4', 3'	5'-OH, 4'		
			OH	5.14 1H		$5'_{a,b}$, 4'		
2'		40.1	a	2.71 1H,	1', 3'	1', 3', $2'_{b}$		
			b	2.27 1H,	4', 3'	1', 3', $2'_{a}$		
2''	A,B,C,D	37.1	a	2.02 1H, brm	$3''_{D}$	$N6_{A,B}$, $1''_{a,b,c,d}$, $3''_{D}$, $2''_{b}$		
		34.4	b	1.80 1H, m	$1''_{D}$, $3''_{B}$	$N6_{A,B}$, $1''_{a,b,c,d}$, $3''_{D}$, $2''_{a}$		

* Overlapped by another peak. See supporting information for D_2O exchange.**Overlapped by 5' signal

Table 2. NMR shifts and correlations for compound **6** in DMSO-d_6.

Figure S1

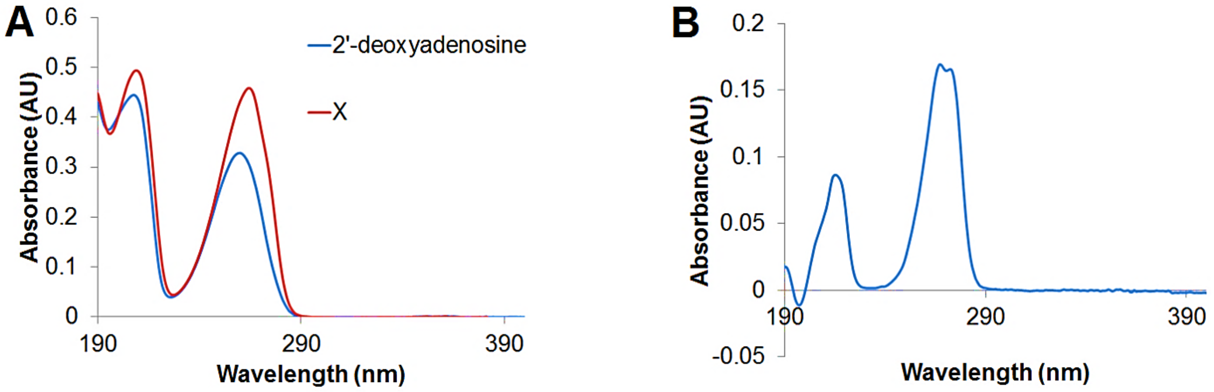

Figure S1. UV-vis spectrum of the cross-link remnant **5**. Panel A: UV absorption spectra for 2'-deoxyadenosine (25 µM) and of **5** (25 µM) in water. Panel B: difference spectra obtained by subtracting the spectra of dA from **5**. Molar absorptivity at 260 nm: ε_{dA} = 13,200 L/(mol·cm), ε_5 = 18,400 L/(mol·cm).

Figure S2

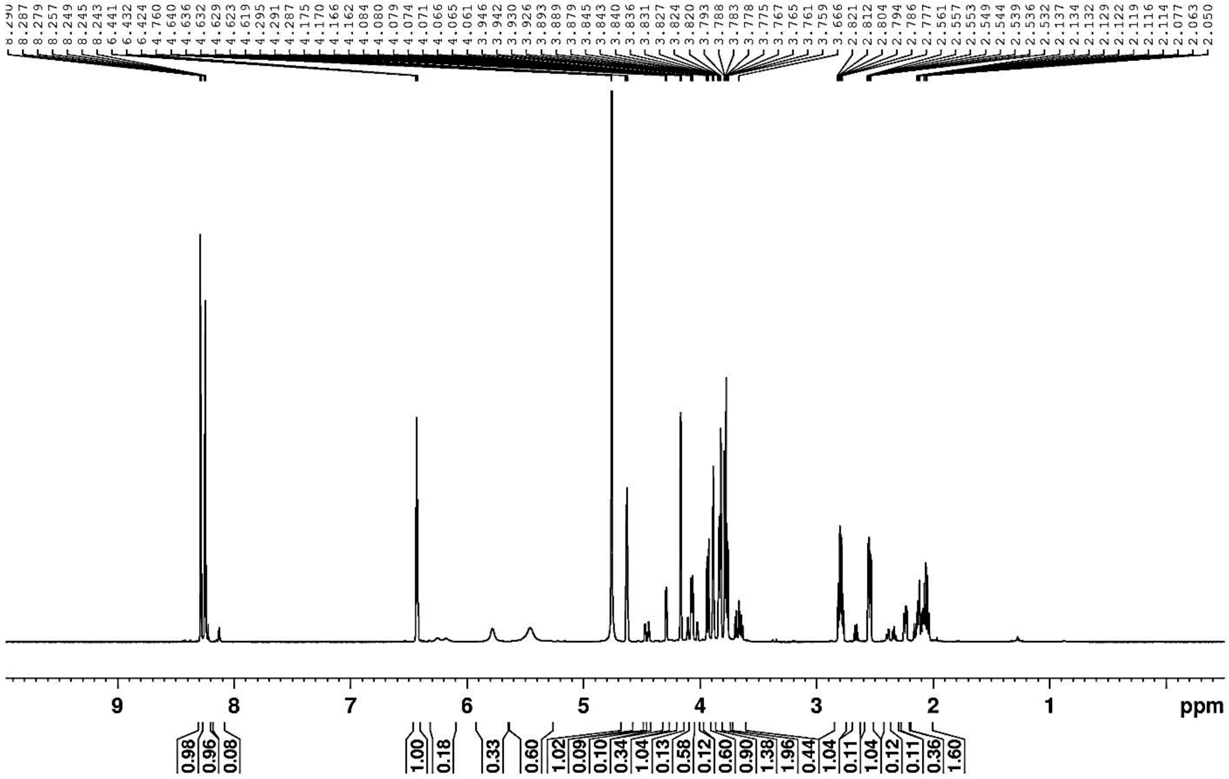

Figure S2. 800 MHz ^{1}H-NMR of **5** in D_2O.

Figure S3

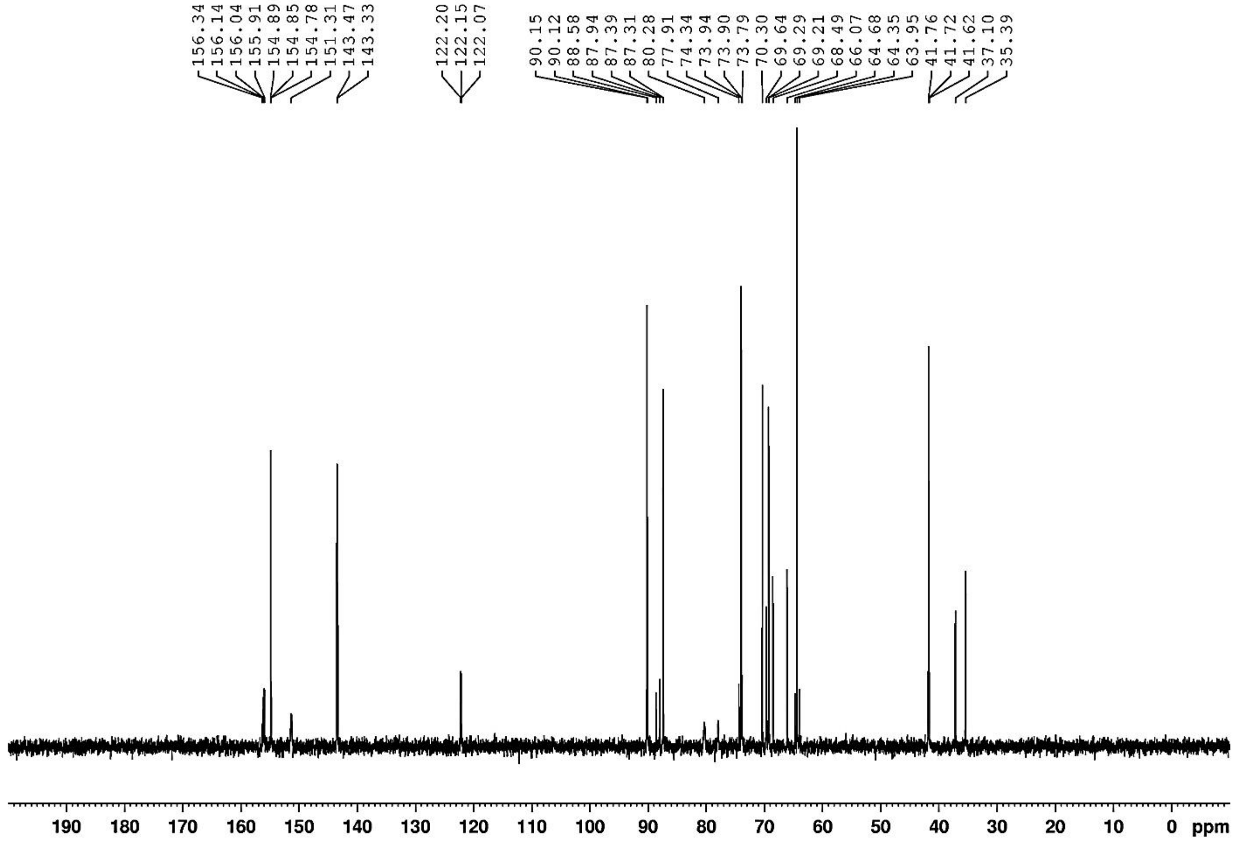

Figure S3. 200 MHz ^{13}C-NMR of **5** in D_2O.

Figure S4

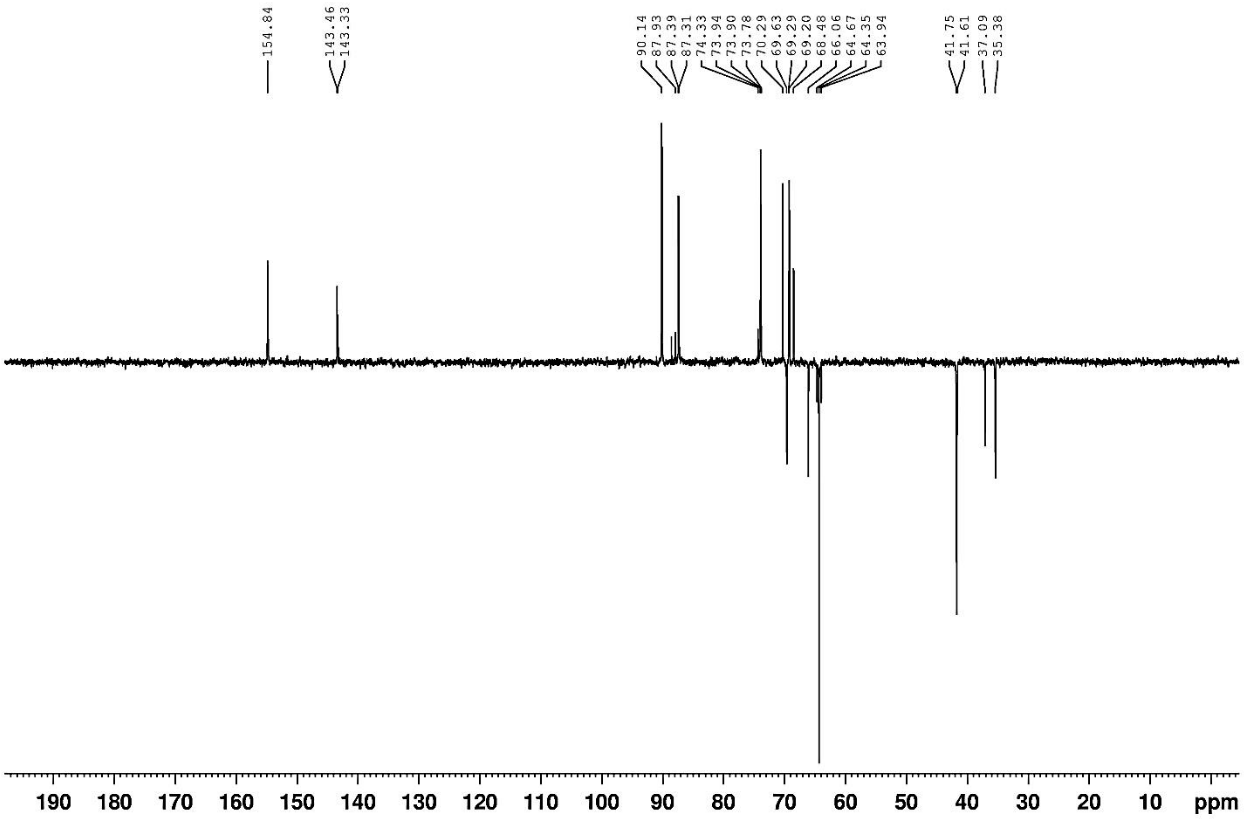

Figure S4. 200 MHz ^{13}C DEPT-NMR of **5** in D_2O.

Figure S5

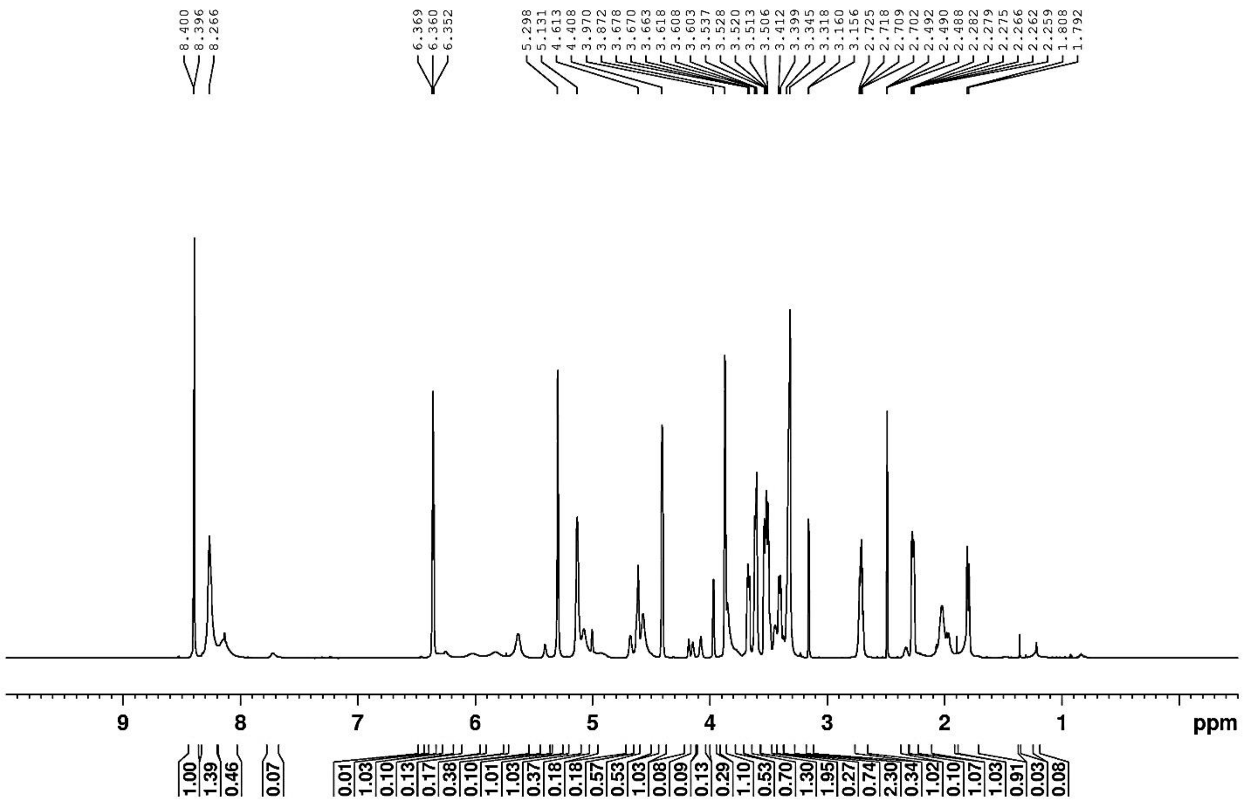

Figure S5. 800 MHz ^{1}H-NMR of **5** in DMSO-(d_6).

Figure S6

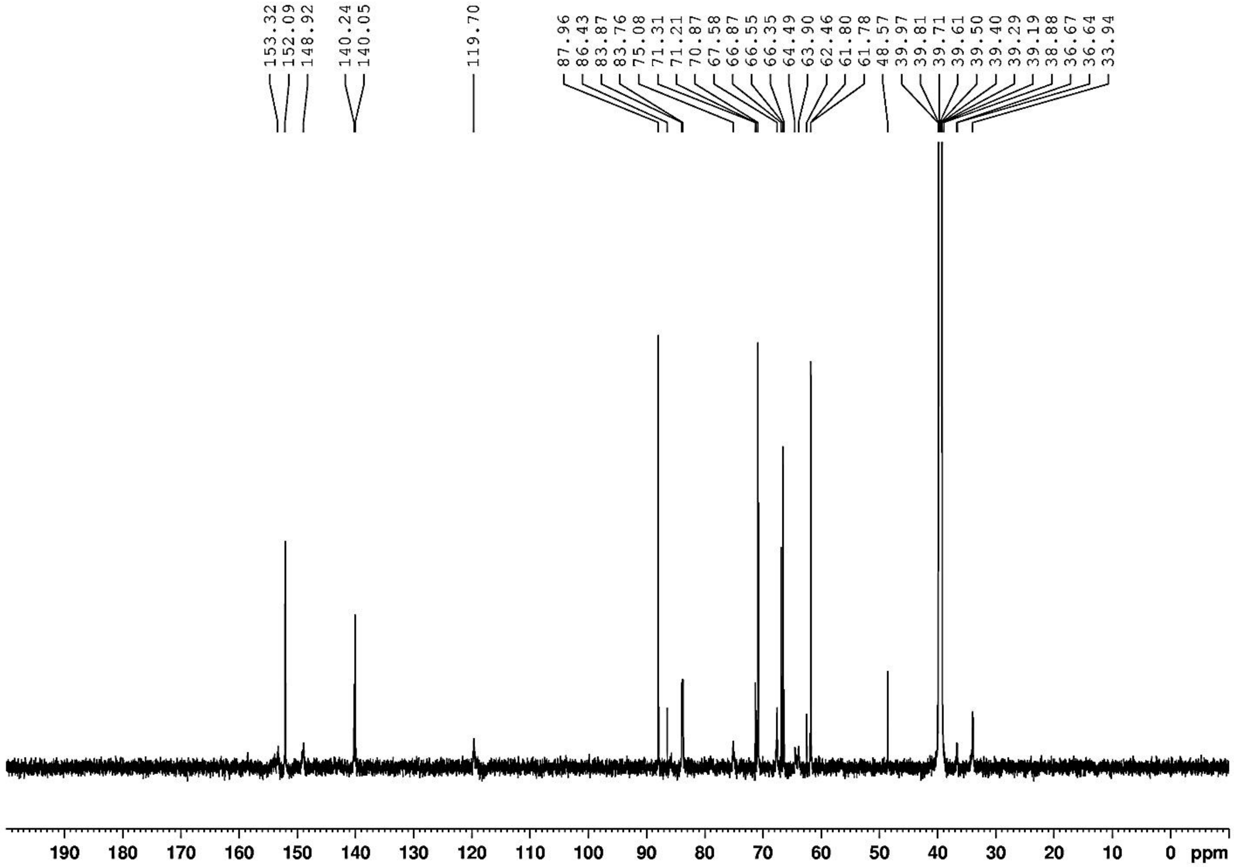

Figure S6. MHz ^{13}C-NMR of **5** in DMSO-(d_6).

Figure S7

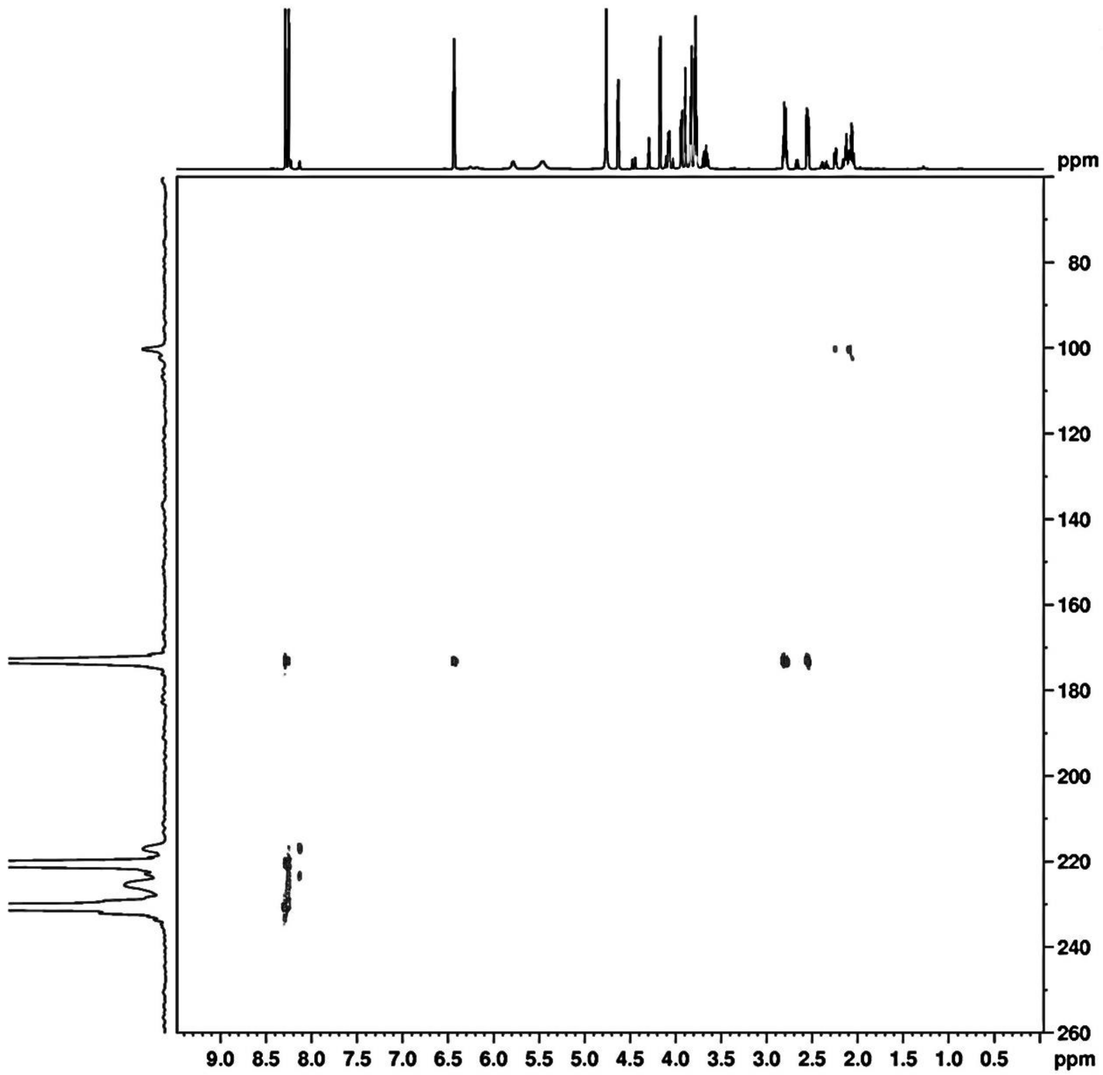

Figure S7. ^{15}N-HMBC of **5** in DMSO-(d_6).

Figure S8

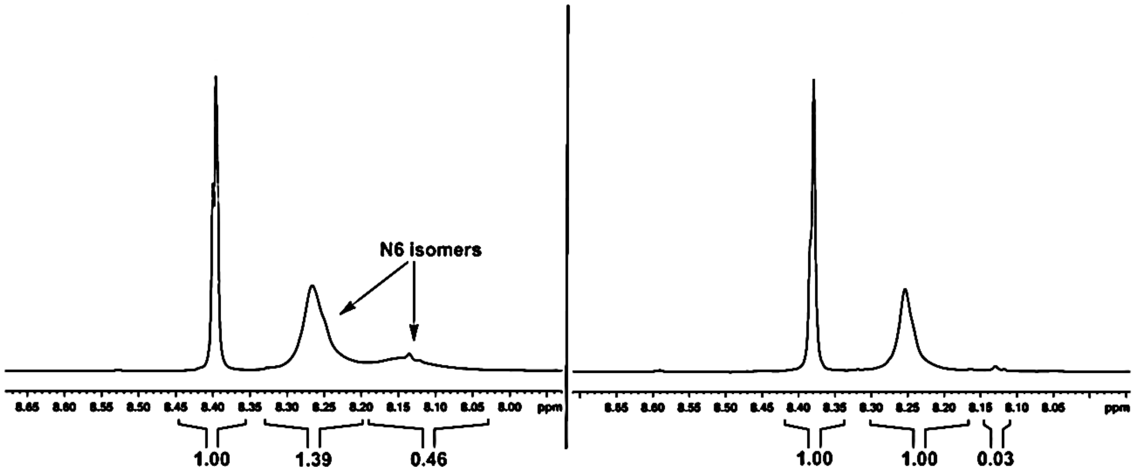

Figure S8. ^{1}H-NMR in DMSO-(d_6) (left) and in DMSO-(d_6) with D_2O exchange (right) of the N^6-protons of **5**. The H2 of dA overlaps with one isomer; however after D_2O exchange the integration is decreased by one proton and the correlations with other peaks also disappear.

Figure S9

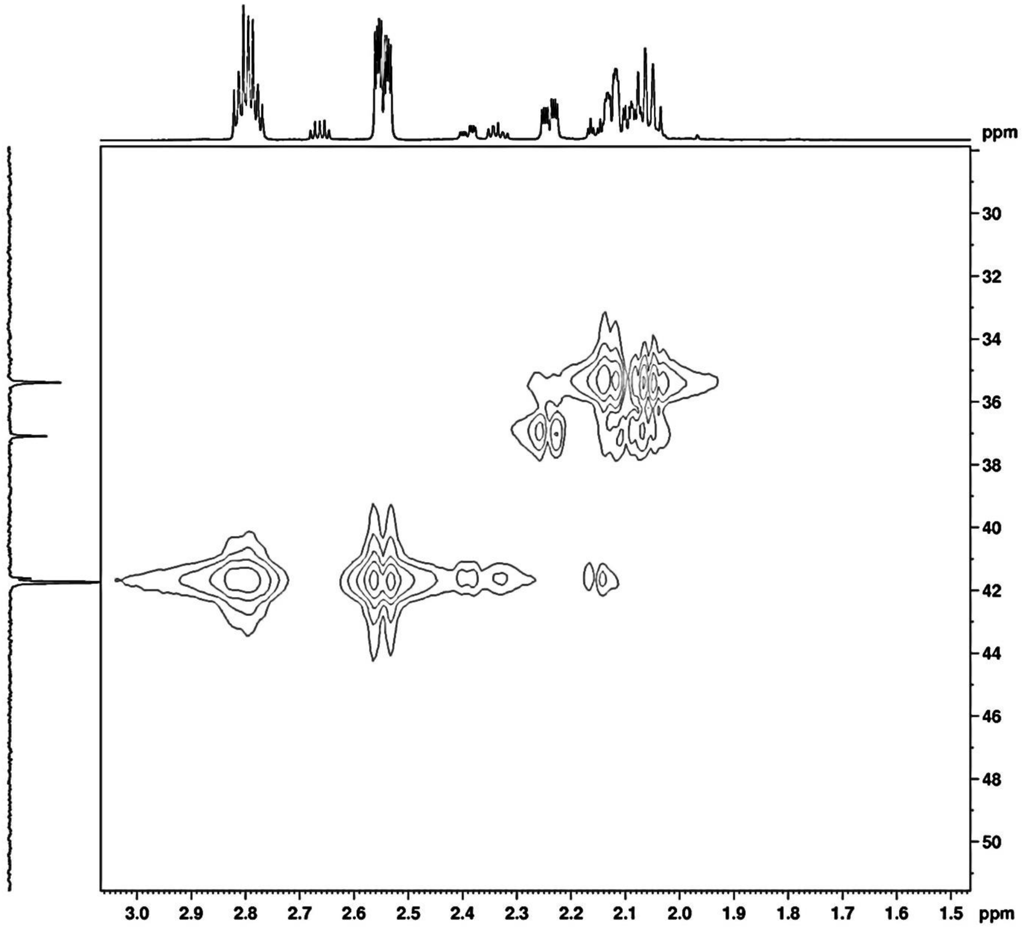

Figure S9. HMQC of the deoxyribose H2' and H2" spectral region of **5** in D_2O.

Figure S10

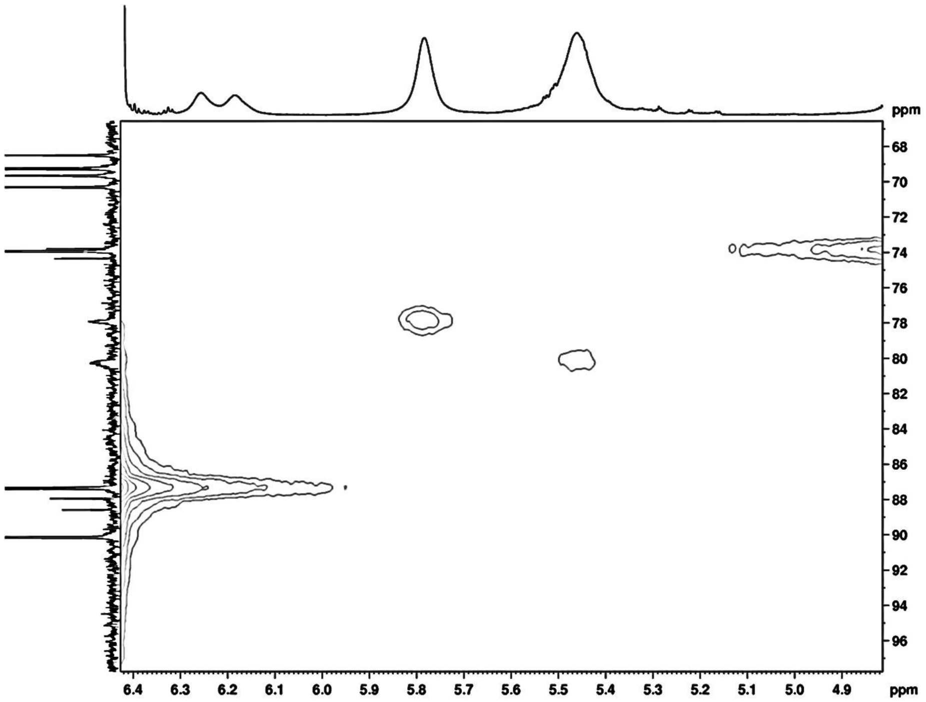

Figure S10. HMQC of the deoxyribose H1' spectral region of **5** in D_2O.

Figure S11

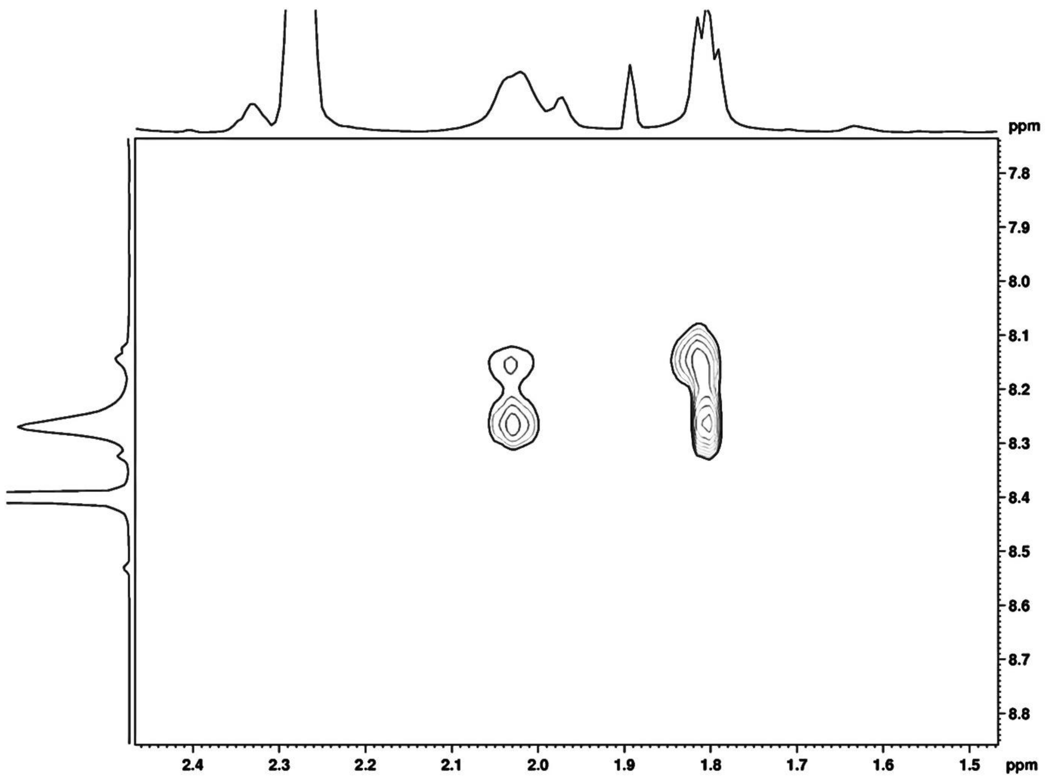

Figure S11. TOCSY of the correlations between N^6-H with H2 hydrogens of the deoxyribose adduct in **5** in DMSO-(d_6).

Figure S12

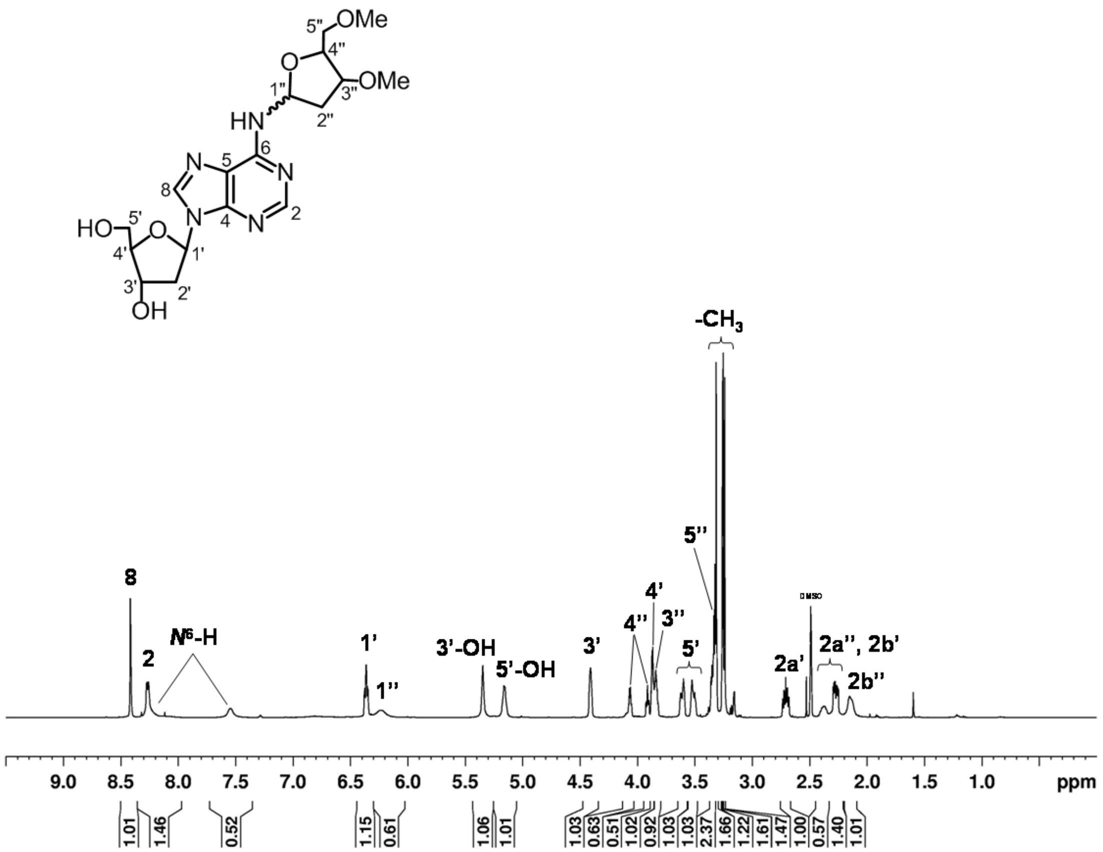

Figure S12. ^{1}H-NMR of **7** in DMSO-(d_6).

Figure S13

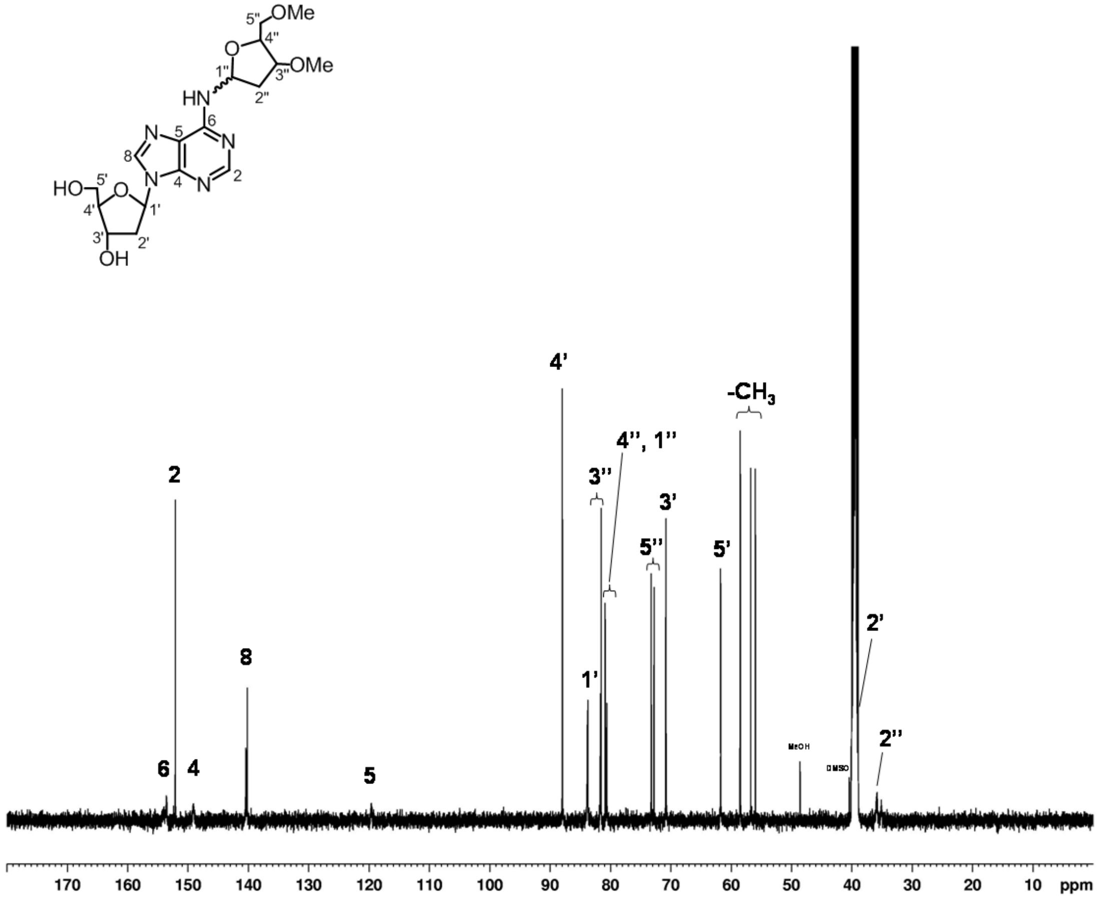

Figure S13. ^{13}C-NMR of **7** in DMSO-(d_6).

Figure S14

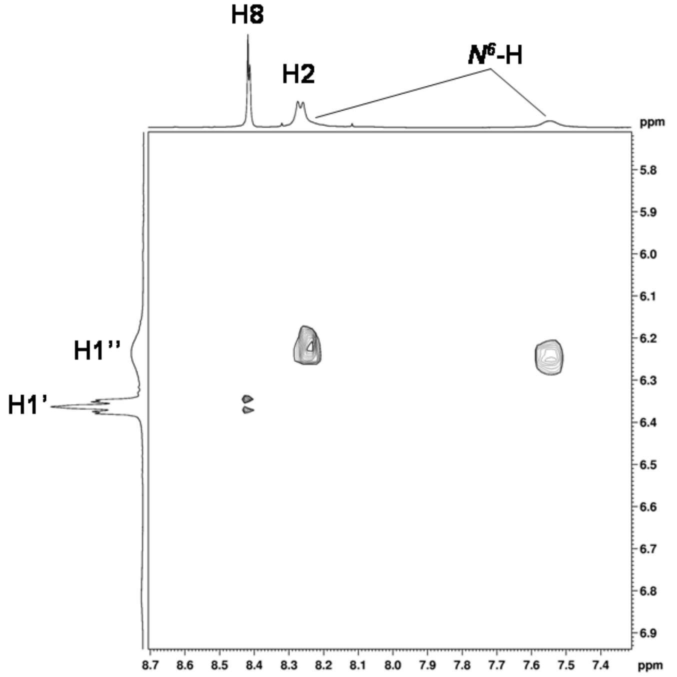

Figure S14. Selected region of the ^{1}H-^{1}H COSY spectrum of **7**. The crosspeaks shown represent homonuclear 3-bond correlations between the exocyclic *N*^{6}-H of dA and H1'' of the 2-deoxyribose adduct.

Figure S15

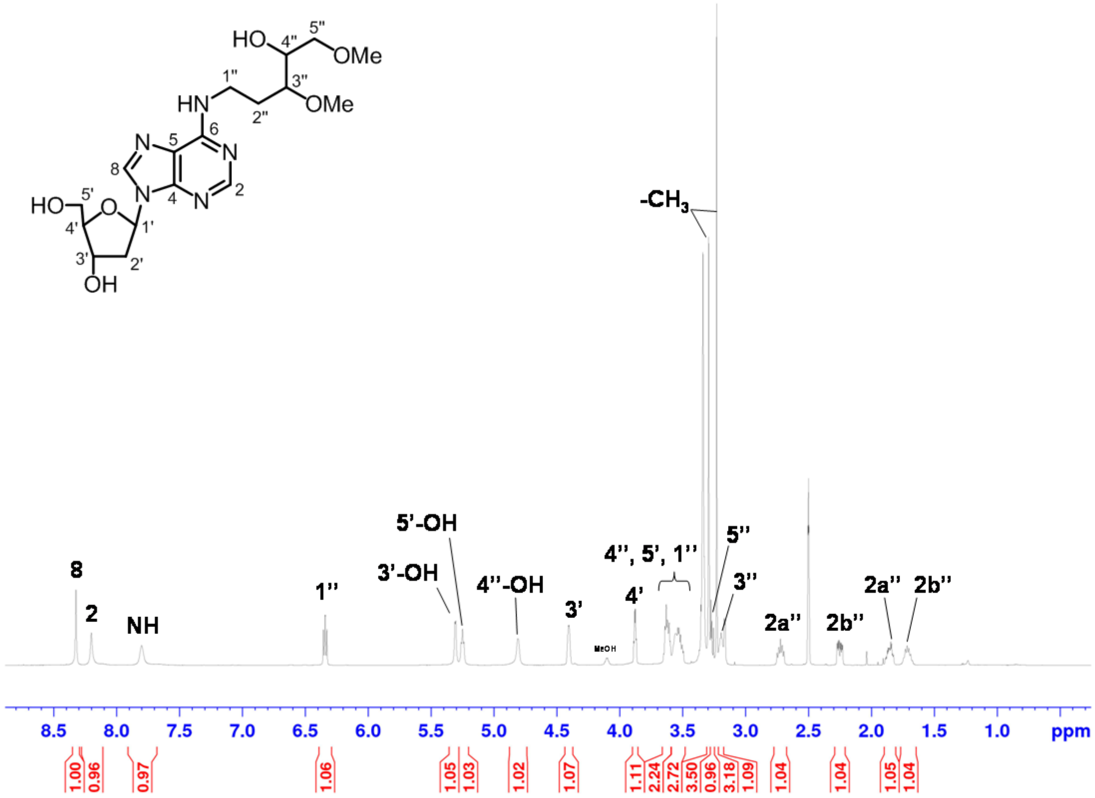

Figure S15. ^{1}H-NMR of **8** in DMSO-(d_6) .

Figure S16

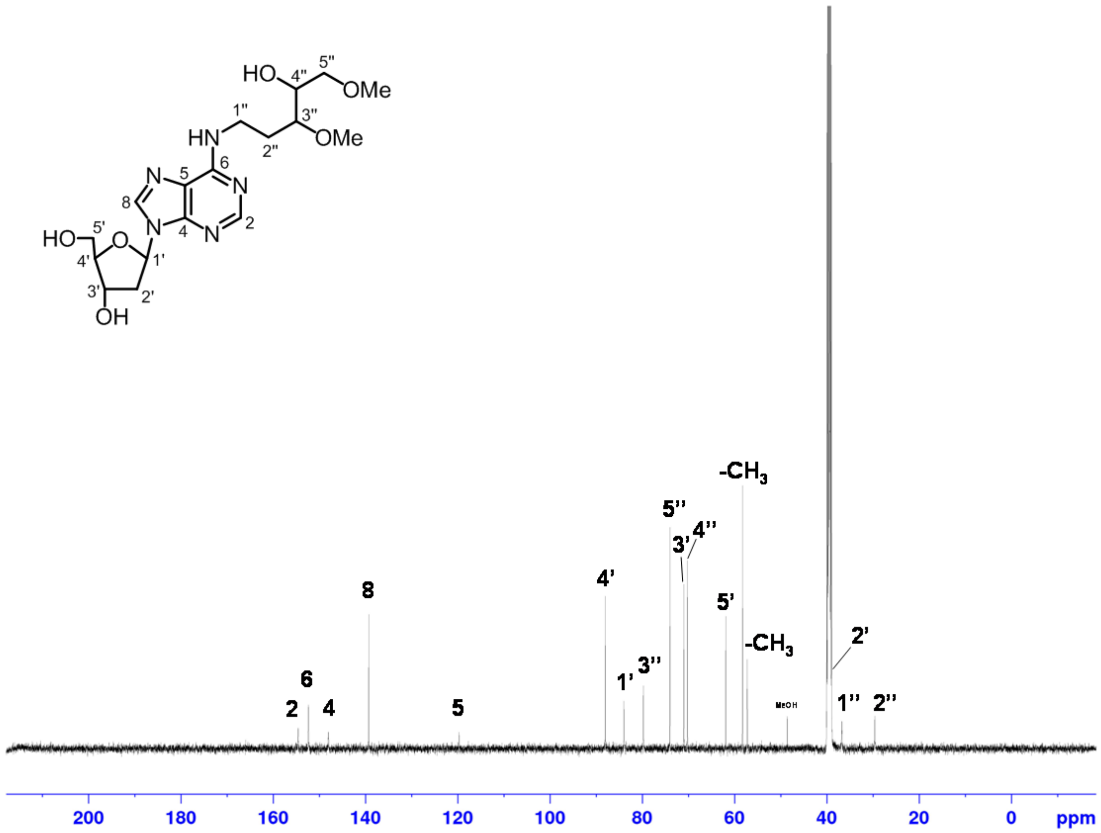

Figure S16. ^{13}C-NMR of **8** in DMSO-(d_6) .

Figure S17

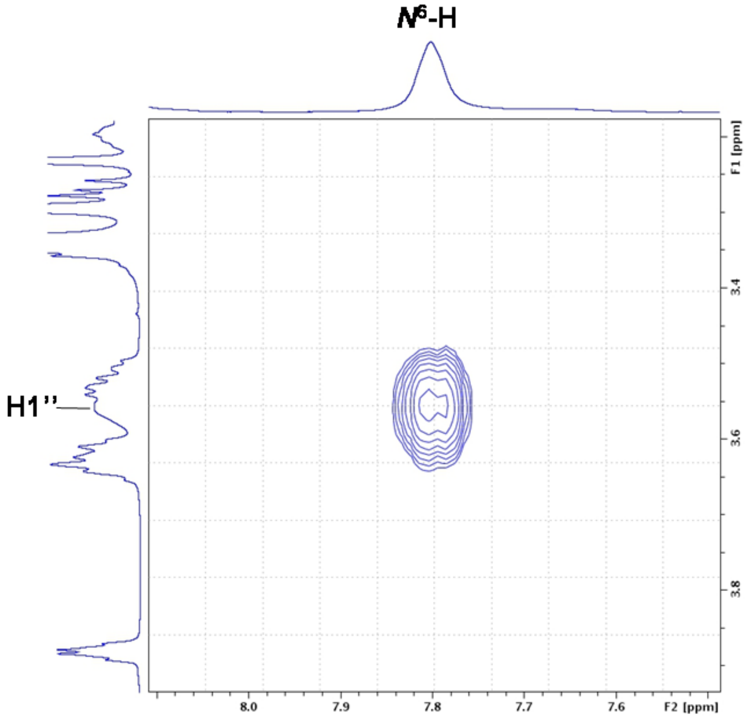

Figure S17. Selected region of ^{1}H-^{1}H COSY spectrum of compound **8**. The crosspeak shown represents the homonuclear 3-bond correlation between the exocyclic N^6-H of dA and H1'' of the reduced 2-deoxyribose adduct.

Figure S18

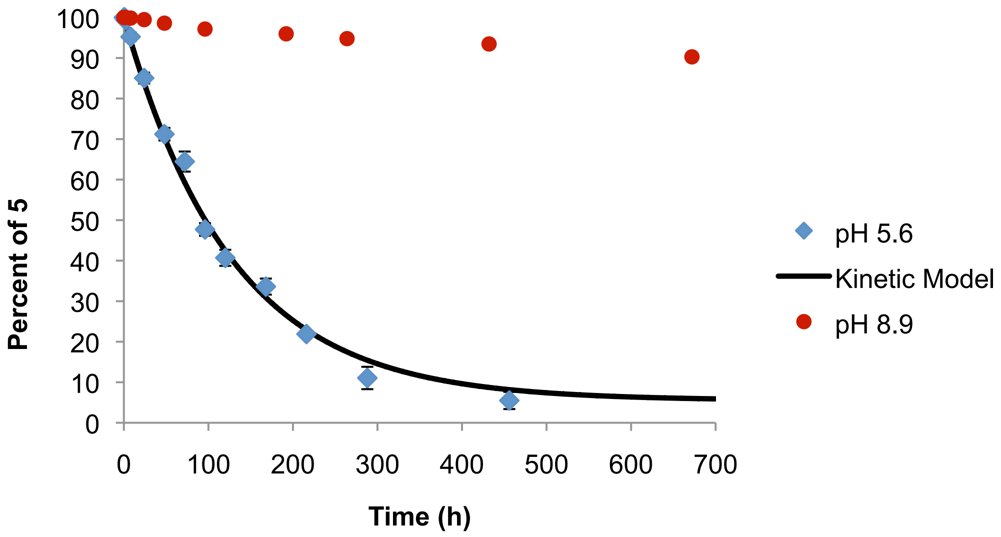

Figure S18. Stability of compound **5** under conditions used to enzymatically digest duplexes **A** and **B**. The blue diamonds represent the disappearance of **5** in pH 5.6 buffer (sodium acetate, 30 mM *erythro*-9-(2-hydroxy-3-nonyl)adenine, 40 µM, $ZnCl_2$, 1 mM, and **5**, 1 mM). The red circles represent the disappearance of **5** in pH 8.9 buffer (Tris-HCl, 100 mM, *erythro*-9-(2-hydroxy-3-nonyl)adenine, 40 µM, $ZnCl_2$, 1 mM, and **5**, 1 mM). The dissociation was measured using the HPLC method described in the Experimental Section of the main body of the paper.

Figure S19

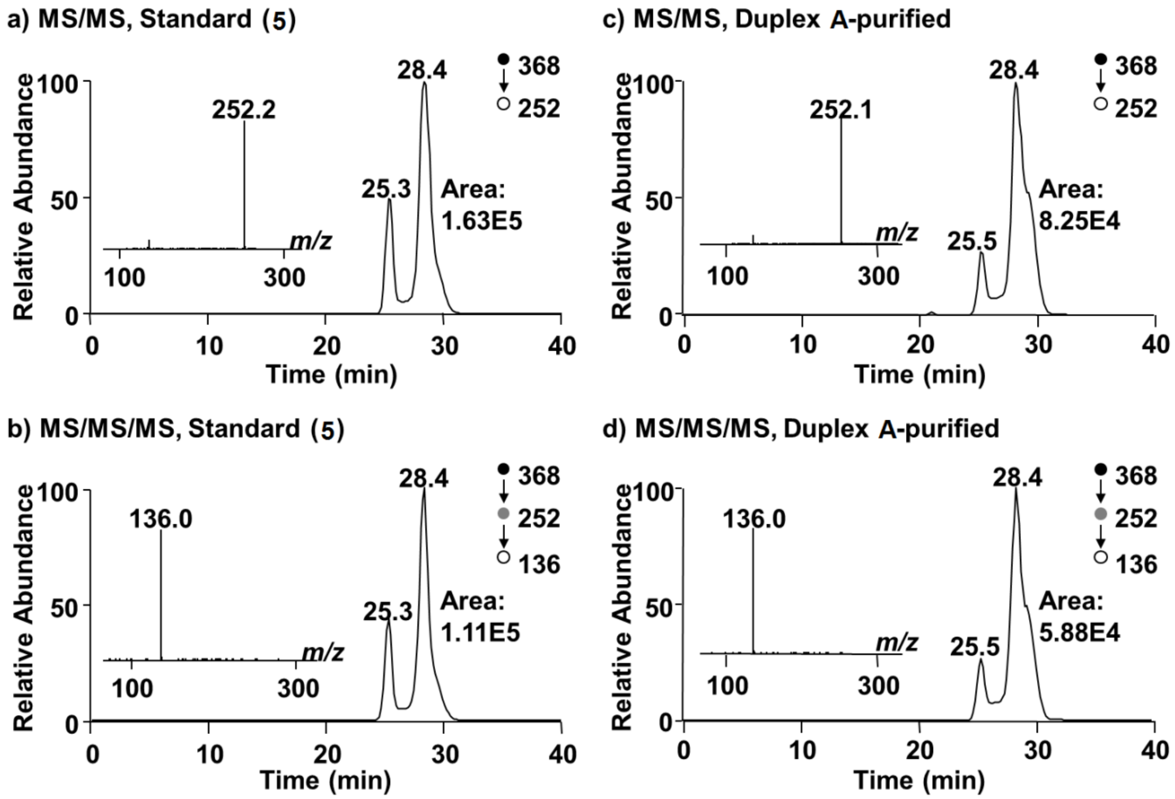

Figure S19. LC-MS/MS of the synthetic standard **5** and the cross-link remnant released by enzymatic digestion of cross-linked duplex **A**. Panels A and B show data for the synthetic nucleoside **5**, while panels C and D show data for the cross-link remnant released by enzymatic digestion of cross-linked duplex **A**. Panels A and C are the chromatograms obtained for the first transition of 368→252. Panels B and D are the chromatograms obtained for the second transition of 252→136.

Figure S20

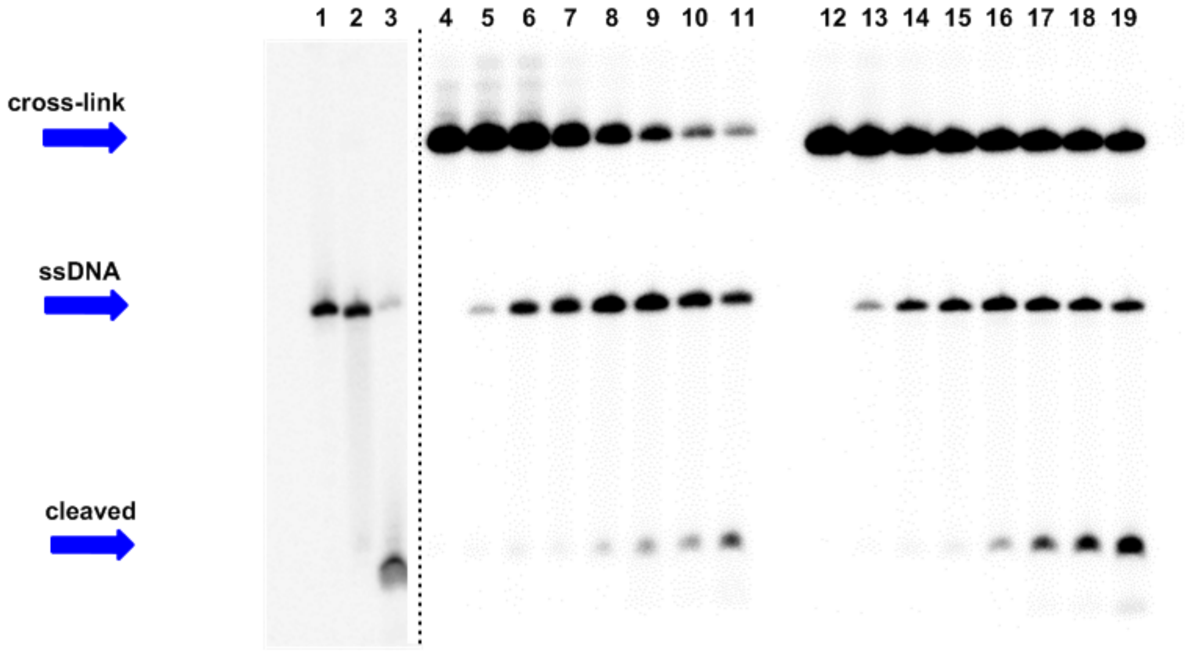

Figure S20. Gel electrophoretic analysis of the stability of the dA-Ap cross-link in ^{32}P-labeled duplexes **A** and **B**. The purified cross-linked duplexes were incubated in HEPES buffer (50 mM, pH 7.0) containing NaCl (100 mM) at 37 °C. Lanes 1-11 show data for duplex **A** and lanes 12-19 depict data for duplex **B**. Lane 1 is a marker lane containing the full-length, 32P-label uracil-containing precursor oligonucleotide from duplex **A**. Lane 2 is the Ap-containing duplex **A**. Lane 3 is the piperidine-treated Ap-containing duplex **A**, showing nearly complete conversion to the 3'-4-hydroxy-2-pentenal-5-phosphate cleavage product. During the incubation, 5 µL aliquots were removed at 0, 0.25, 1, 2, 5, 10, 15, and 21 days, the DNA ethanol precipitated, and the DNA stored at –20 ˚C until prior to analysis on by 20% denaturing polyacrylamide gel electrophoresis. Lanes 4-11 show the time-course for dissociation of the cross-link in duplex **A**, while lanes 12-19 show the time course for the dissociation of the cross-link in duplex **B**. The amount of DNA in each band was quantitatively measured by phosphorimager analysis.

Figure S21

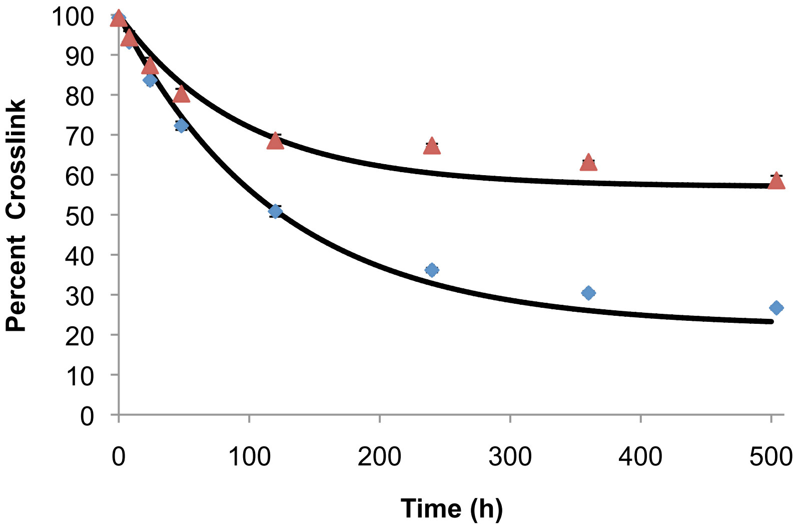

Figure S21. A plot of the gel electrophoretic data shown in Figure S20, monitoring the stability of the dA-Ap cross-link in duplexes **A** and **B** incubated in HEPES buffer (50 mM, pH 7.0) containing NaCl (100 mM) at 37 °C. The red triangles show the data for duplex **B** and blue diamonds show the data for duplex **A**. The data was fit to a first-order decay to give apparent first-order rate constants of k = 8.2 x 10^{-3} h^{-1} for the dissociation of duplex **A** and k = 1.1 x10^{-2} h^{-1} for duplex **B**. The corresponding half-lives of dissociation are 84 h for duplex **A** and 66 hours for duplex **B**.

Figure S22

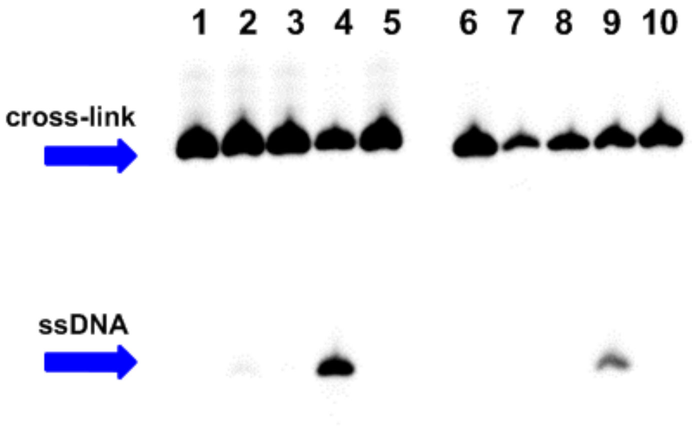

Figure S22. 20% Denaturing polyacrylamide gel electrophoretic analysis of the stability of the dA-Ap cross-link in ^{32}P-labeled duplexes **A** and **B** incubated for 96 h at various temperatures and pH values. Lanes 1-5 show the purified duplex **A** subjected to various conditions. Lane 1: isolated cross-link standard. Lane 2: sodium phosphate (20 mM, pH 7) containing NaCl (100 mM) at 22 °C for 96 h, Lane 3: sodium phosphate (20 mM, pH 7) containing NaCl (100 mM) at 4 °C for 96 h, Lane 4: sodium acetate (20 mM, pH 5.2) containing NaCl (100 mM) at 22°C for 96 h, and Lane 5: Tris-borate buffer (13 mM and 4 mM, respectively, pH 9.2) containing NaCl (100 mM) at 22°C for 96 h. Lanes 6-10 show data for duplex **B**. Lane 6: isolated cross-link standard. Lane 7: sodium phosphate (20 mM, pH 7) containing NaCl (100 mM) at 22 °C for 96 h, Lane 8: sodium phosphate (20 mM, pH 7) containing NaCl (100 mM) at 4 °C for 96 h, Lane 9: sodium acetate (20 mM, pH 5.2) containing NaCl (100 mM) at 22°C for 96 h, and Lane 10: Tris-borate buffer (13 mM and 4 mM, respectively, pH 9.2) containing NaCl (100 mM) at 22°C for 96 h. The intensity of each band was quantitatively measured by phosphorimager analysis.
